# BEAN and HABAS: Polyphyletic insertions in the DNA‐directed RNA polymerase

**DOI:** 10.1002/pro.5194

**Published:** 2024-10-28

**Authors:** Claudia Alvarez‐Carreño, Angela T. Huynh, Anton S. Petrov, Christine Orengo, Loren Dean Williams

**Affiliations:** ^1^ Institute of Structural and Molecular Biology University College London London UK; ^2^ School of Chemistry and Biochemistry Georgia Institute of Technology Atlanta Georgia USA; ^3^ NASA Center for the Origin of Life Georgia Institute of Technology Atlanta Georgia USA

**Keywords:** bacteria, insertional domains, protein evolution, transcription

## Abstract

The β and β′ subunits of the RNA polymerase (RNAP) are large proteins with complex multi‐domain architectures that include several insertional domains. Here, we analyze the domain organizations of RNAP‐β and RNAP‐β′ using sequence, experimentally determined structures and AlphaFold structure predictions. We observe that lineage‐specific insertional domains in bacterial RNAP‐β belong to a group that we call BEAN (broadly embedded annex). We observe that lineage‐specific insertional domains in bacterial RNAP‐β′ belong to a group that we call HABAS (hammerhead/barrel‐sandwich hybrid). The BEAN domain has a characteristic three‐dimensional structure composed of two square bracket‐like elements that are antiparallel relative to each other. The HABAS domain contains a four‐stranded open β‐sheet with a GD‐box‐like motif in one of the β‐strands and the adjoining loop. The BEAN domain is inserted not only in the bacterial RNAP‐β′, but also in the archaeal version of universal ribosomal protein L10. The HABAS domain is inserted in several metabolic proteins. The phylogenetic distributions of bacterial lineage‐specific insertional domains of β and β′ subunits of RNAP follow the Tree of Life. The presence of insertional domains can help establish a relative timeline of events in the evolution of a protein because insertion is inferred to post‐date the base domain. We discuss mechanisms that might account for the discovery of homologous insertional domains in non‐equivalent locations in bacteria and archaea.

## 1 INTRODUCTION

Transcription is the Central Dogma process in which RNA polymerase (RNAP) transcribes DNA into RNA (Hurwitz et al., 1961). mRNA is then translated into protein in the ribosome. RNAP contains five subunits called α1, α2, β, β′, and ϖ. The β and β′ subunits of RNAP, the focus of this work, both contain double‐Ψ‐β‐barrel (DΨBB) domains, which combine to form the catalytic core of RNAP (Castillo et al., 1999; Iyer et al., 2003). RNAP‐β and RNAP‐β′are large proteins with complex multi‐domain architectures.

RNAP‐β and RNAP‐β′ have bacterial, archaeal, and eukaryotic orthologs, with sequence motifs and domains that are universal over the tree of life (Jokerst et al., 1989; Lane & Darst, 2010a, 2010b; Sweetser et al., 1987). However, the domain architectures of RNAP‐β and RNAP‐β′ vary significantly between archaea and bacteria, and among bacteria. Archaea‐specific domains of RNAP are conserved in eukaryotes (Figures S1 and S2). Interestingly, some bacteria‐specific domains of RNAP are observed only in certain bacterial lineages (Borukhov et al., 1991; Huang et al., 2015; Iyer et al., 2004; Lane & Darst, 2010a, 2010b; Qayyum et al., 2024; Severinov et al., 1992).

Proteins most commonly acquire new domains by terminal addition (Marsh & Teichmann, 2010; Weiner et al., 2006), generating tandem multidomain architectures. Yet, both RNAP‐β and RNAP‐β′ have acquired domains by internal insertion, generating discontinuous domain architectures. In general, insertional domains are less frequent than terminally‐added domains (Manriquez‐Sandoval & Fried, 2022). In bacterial RNAPs, insertional domains have accreted within preexisting base domains, even within other insertional domains. These insertional accretion processes cause dependencies that can be particularly useful in understanding chronological ordering of domain accumulation: an insertional domain perches on a base domain, thus, indicating that the insertional domain was acquired more recently than the base domain.

Here, we use a naming scheme in which domains of RNAP‐β and RNAP‐β′ that occur in all archaea but not in bacteria are “a‐specific” domains. Domains that occur in all bacteria but not in archaea are “b‐specific.” Domains that occur in some bacterial lineages but not others are “b/lineage‐specific.”

We use sequences and structures to reconstruct an extraordinary succession of events that occurred in the deep evolutionary history of RNAP. Our analysis shows that bacterial lineages acquired specific types of insertional domains at multiple locations of RNAP‐β and RNAP‐β′. Archaeal lineages acquired different insertional domains. Specifically, we identify a broadly distributed b/lineage‐specific insertional domain with idiosyncratic positions in RNAP‐β. We call this domain BEAN (broadly embedded annex). The BEAN domain is also identified in the bacterial RNAP‐β′ and in the archaeal version of universal ribosomal protein L10 (uL10). We identify a b/lineage‐specific insertional domain with idiosyncratic position in RNAP‐β′. We call this domain HABAS (hammerhead/barrel‐sandwich hybrid). The HABAS domain is also observed in bacterial RNAP‐β and as an insertional domain in several metabolic proteins. Our results, lead naturally to a classification system for bacterial RNAP‐β and RNAP‐β′ subunits, based on type, location and chronology of domain insertion.

We describe extensive insertional diversity with the DST (deinococcus‐thermus, synergistetes, thermotogae and related bacteria) group.

## 2 RESULTS

### 2.1 Domain organizations of RNAP β and β′

**FIGURE 1:**
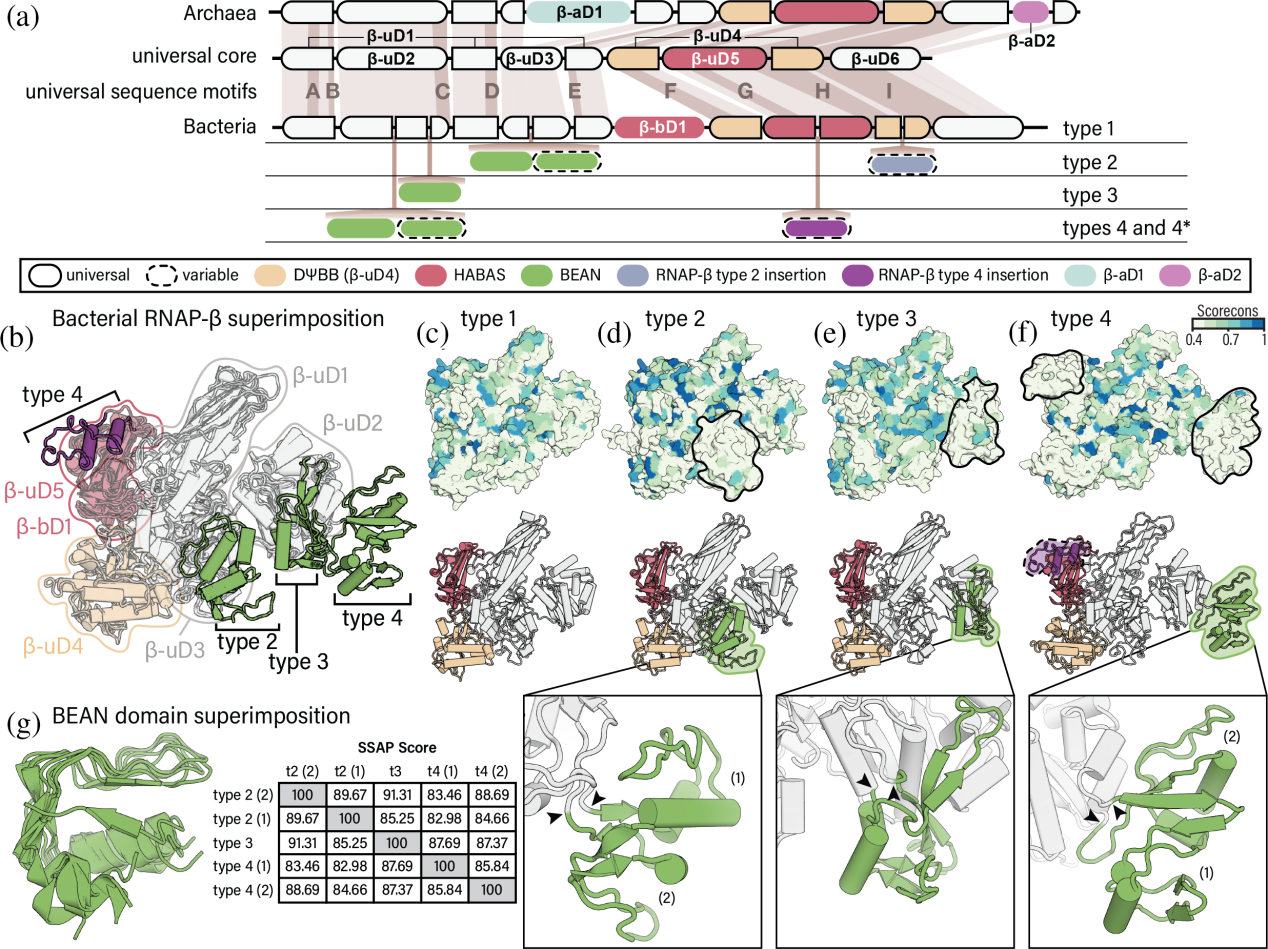
Domain organization of RNAP‐βs. (a) RNAP‐β in archaea and bacteria. First row: Domains of archaeal RNAP‐β. Second row: Universal domains of RNAP‐β shared between archaeal and bacterial orthologs and universal sequence motifs described in Sweetser et al. (1987). Third row: Domains of bacterial type 1 RNAP‐β. Fourth row: Location of bacterial type 2 insertions. Fifth row: Location of bacterial type 3 insertions. Sixth row: Location of bacterial type 4 insertions. (b) Superimposition of bacterial RNAP‐β structures. (c) type 1 RNAP‐β (AlphaFold DB: AF‐A2BT61‐F1); (d) type 2 (AlphaFold DB: AF‐A9B6J3‐F1); (e) type 3 (AlphaFold DB: AF‐Q8ETY8‐F1) and (f) type 4 (PDB: 4IGC, chain C). In panels c to f, RNAP‐β structures are colored by degree of sequence conservation (upper panel) and by domain (lower panel). The insets show detailed views of BEAN domain insertions. The sites of insertion are marked by black arrowheads. The conservation score ranges from 0 (not conserved) to 1 (highly conserved). Valdar01 scores were calculated on the multiple sequence alignment of representatives for each type using Scorecons. For clarity, N‐ and C‐terminal residues that extend beyond the shared core of bacteria are masked. (g) Structure superimposition of type‐specific BEAN domains in RNAP‐β and pairwise SSAP (sequential structure alignment program) score. Only the core secondary structural elements are shown. The SSAP score ranges from 0 to 100. Scores above 70 indicate similar folds. Domain labels and colors are consistent throughout all panels.

**FIGURE 2:**
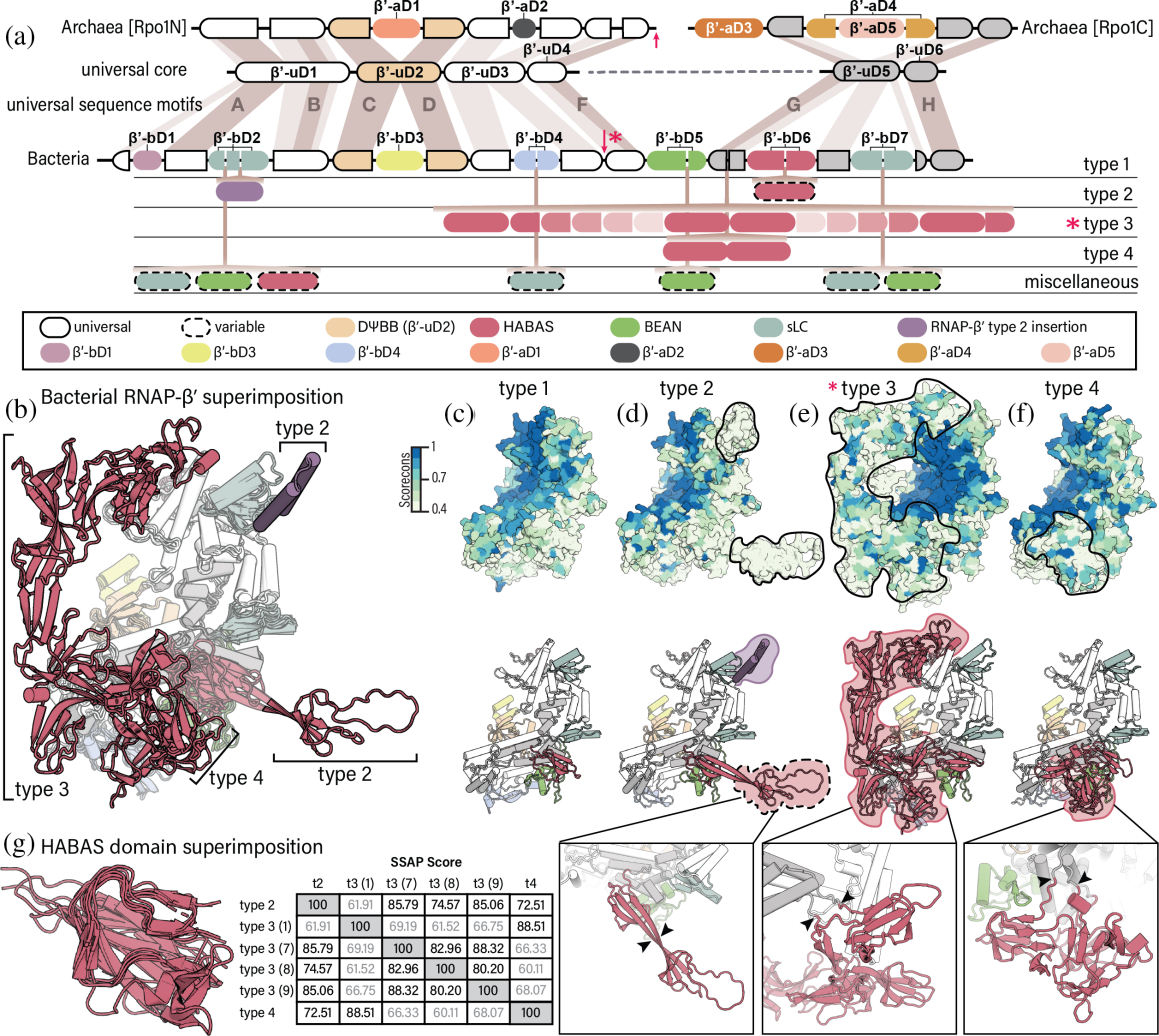
Domain organization of RNAP‐β′s. (a) RNAP‐β′ in archaea and bacteria. First row: Domains of archaeal orthologs. Second row: Universal domains shared between archaeal and bacterial orthologs and universal sequence motifs described in Jokerst et al. (1989). Third row: Domain organization of the bacterial type 1 RNAP‐β′. Fourth row: Location of bacterial type 2 insertions. Fifth row: Location of bacterial type 3 insertions. Sixth row: Location of bacterial type 4 insertions. Red arrows: Sites of split of RNAP‐β′ into sub‐subunits. Red asterisk: Truncation in bacteria is observed only in type 3 RNAP‐β. (b) Superimposition of bacterial RNAP‐β′ structures. (c) type 1 (AlphaFold DB: AF‐Q0AUH3‐F1), (d) type 2 (AlphaFold DB: AF‐Q3Z8V3‐F1), (e) type 3, N‐terminal fragment (AlphaFold DB: AF‐A2BT60‐F1) and C‐terminal fragment (AlphaFold DB: AF‐A2BT59‐F1), and (f) type 4 (AlphaFold DB: AF‐A7IKQ1‐F1). In panels c to f, RNAP‐β′ structures are colored by conservation (upper panel) and by domain (lower panel). The insets show detailed views. Black arrowheads indicate the sites of insertion of type‐specific HABAS domains. The conservation score ranges from 0 (not conserved) to 1 (highly conserved). Valdar01 scores were calculated on the multiple sequence alignment of sequence representatives for each type using Scorecons. For clarity, N‐ and C‐terminal residues that extend beyond the shared core of bacteria were masked. (g) Structure superimposition of type‐specific HABAS domains in RNAP‐β′ and pairwise SSAP (sequential structure alignment program) score. The SSAP scores range from 0 to 100. Scores above 70 indicate similar fold. SSAP scores below 70 are shown in gray. Domain are labels and colors are consistent throughout all panels.

We analyzed the domain structures of RNAP‐β and RNAP‐β′ using orthologous sequences from a subsample of a reference set of evenly sampled bacterial genomes (Zhu et al., 2019). The subsample used here includes representatives from all known major bacterial species and has been adapted from Moody et al. (2022). Multiple sequence alignments (MSAs) of RNAP‐β and RNAP‐β′ display block structures indicating universal as well as a‐specific, b‐specific and b/lineage‐specific sequences (Figures S1a and S2a). Most a‐, b‐ and b/lineage‐specific domains of RNAP‐β and RNAP‐β′ are insertional (Figures 1a and 2a).

The sequence blocks within MSAs were annotated using CATH (Sillitoe et al., 2021) (Tables 1 and 2). The location and type of insertion were verified using experimentally determined structures (Berman et al., 2000) and AlphaFold structure predictions (Jumper et al., 2021; Varadi et al., 2022). We observe small insertions (<50 residues) that lack sequence similarity to each other or to domain entries in three classification databases: CATH (Sillitoe et al., 2021), ECOD (Schaeffer et al., 2017) and SCOPe (Chandonia et al., 2017). These insertions are omitted from the analysis here.

**TABLE 1:** Multi‐domain architecture of RNAP β subunit in representatives from archaea and bacteria.

Short name	CATH ID	Archaea	Bacterial type 1	Bacterial type 2	Bacterial type 3	Bacterial type 4
		P11513 (AAY80073.1)	A2BT61 (ABM70972.1)	A9B6J3 (ABX02896.1)	Q8ETY8 (BAC12068.1)	P0A8V4 (AAC76961.1)
		*Sulfolobus acidocaldarius*	*Prochlorococcus marinus*	*Herpetosiphon aurantiacus*	*Oceanobacillus iheyensis*	*Escherichia coli*
β‐uD1	3.90.1100.10	1–166; 338–476	1–132; 315–442; 524–581	1–159; 364–491; 662–719	1–142; 406–535; 618–675	1–152; 450–577; 675–714
β‐uD2	3.90.1110.10	167–337	133–314	160–363	143–279; 371–407	153–226; 343–449
Type 3 insertion	3.90.105.10	‐	‐	‐	286–370	‐
Type 4 insertion	3.90.105.10	‐	‐	‐	‐	240–338
β‐uD3	2.30.150.10	471–476; 569–711	443–523	492–510; 602–661	536–617	578–674
β‐aD1	3.90.1070.20	477–568	‐	‐	‐	‐
Type 2 insertion	3.90.105.10	‐	‐	511–601	‐	‐
β‐bD1	2.40.50.100	‐	582–650	720–788	676–752	715–790
β‐uD4	2.40.270.10	712–747; 869–994	651–687; 819–886; 887‐913; 916‐923; 925–958	789–825; 957–1081	753–789; 921–1045	791–827; 1059–1237
β‐uD5	2.40.50.150	748–868	688–818	826–956	790–920	828–939; 1038–1058
Type 4 insertion	6.10.140.1670	‐	‐	‐	‐	940–1037
β‐uD6	3.90.1800.10	995–1130	959–1077	1082–1220	1046–1180	1238–1400

**TABLE 2:** Multi‐domain architecture of RNAP β′ subunit in representatives from archaea and bacteria.

Short name	CATH	Archaea	Bacterial type 1	Bacterial type 2	Bacterial type 3 (N‐ter)	Bacterial type 3 (C‐ter)	Bacterial type 4
		P11512 (AAY80072.1)	Q0AUH3 (ABI69631.1)	Q3Z8V3 (AAW40096.1)	A2BT60 (ABM70971.1)	A2BT59 (ABM70970.1)	A7IKQ1 (ABS68594.1)
		*Sulfolobus acidocaldarius*	*Syntrophomonas wolfei* subsp. *wolfei*	*Dehalococcoides mccartyi*	*Prochlorococcus marinus*	*Prochlorococcus marinus*	*Xanthobacter autotrophicus*
β′‐uD1	4.10.960.120	1–148; 176–317	10–64; 93–138; 178–343	1–56; 84–130; 237–402	1–70; 98–144; 192–357	‐	1–67; 95–141; 182–348
β′‐bD1	3.90.820.30	‐	65–91	57–83	71–97	‐	68–94
Archaea‐specific insertion	‐	149–175	‐	‐	‐	‐	‐
β′‐bD2	3.30.60.280	‐	139–177	131–140; 211–236	145–191	‐	142–181
Type 2 insertion	‐	‐	‐	141–210	‐	‐	‐
β′‐uD2	2.40.40.20	318–346; 414–494	344–371; 414–495	403–430; 475–554	358–385; 428–509	‐	349–376; 419–500
β′‐bD3	1.10.40.90	‐	372–413	431–474	386–427	‐	477–418
β′‐aD1	3.30.1490.180	347–413	‐	‐	‐	‐	‐
β′‐uD3	1.10.274.100	495–560; 599–644	496–512; 579–640	555–571; 618–679	510–526; 618–635	‐	501–517; 572–637
β′‐bD4	‐	‐	513–578	572–617	527–617	1–84	518–571
β′‐aD2	2.60.40.2940	561–598	‐	‐	‐	‐	‐
β′‐uD4	1.10.132.30	645–832	641–798	680–837	‐	85–244	638–806
		1054–1093					
Archaea‐sepecific insertion	‐	756–779	‐	‐	‐	‐	‐
β′‐bD5	3.90.105.10	‐	799–879	838–917	‐	245–318	807–888
β′‐uD5	1.10.1790.20	‐	880–949; 1013–1059; 1104–1113	918–987; 1118–1164; 1209–1282	‐	319–369; 1009–1027; 1092–1154; 1199–1208	889–939; 1124–1143; 1208–1254; 1299–1308
β′‐bD6	2.40.50.100	‐	950–1012	988–1015; 1085–1117	‐	1028–1091	1144–1207
Type 2 insertion				1016–1086			
Type 3 insertion	2.40.50.100	‐	‐	‐	‐	370–1008*	‐
Type 4 insertion	1.10.132.30	‐	‐	‐	‐	‐	940–1123*
β′‐bD7	3.30.60.280	‐	1060–1103	1165–1208	‐	1155–1198	1255–1298
β′‐uD6	1.10.150.390	‐	1114–1181	1219–1283		1209–1252	1309–1375

Our investigation here is facilitated by our naming scheme for RNAP domains. In this scheme, the subunit is indicated by β or β′, followed by a hyphen and the letter “u” to indicate universal conservation, or “a” to indicate a‐specific, or “b” to indicate b‐specific. The domains (D) are numbered in order of appearance in the sequence. For example, the N‐terminal RNAP β‐subunit domain, which is universal, is called β‐uD1.

In Candidatus Adlerbacteria and *Wolinella succinogenes* the β and β′ subunits are fused into one polypeptide chain (Table S1). For our analysis, these polypeptides were split into two chains, based on homology.

### 2.2 RNAP‐β multi‐domain architectures

RNAP‐β contains six domains that are conserved in archaeal and bacterial and eukaryotic orthologs (i.e., are universal, Figures 1a and S1a). These universal domains are β‐uD1, β‐uD2, β‐uD3, β‐uD4, β‐uD5, and β‐uD6. Three of these universal domains are insertional: β‐uD2 is inserted into β‐uD1; β‐uD3 is inserted into β‐uD1; and β‐uD5 (a HABAS domain) is inserted into β‐uD4 (the DΨBB domain).

Archaea and bacteria RNAP‐βs each contain additional domains (Figures 1a and S3). In archaea, β‐aD1 is inserted within β‐uD3 and β‐aD2 is inserted within β‐uD6. In bacteria, β‐bD1 is inserted between β‐uD2 and β‐uD4.

**FIGURE 3:**
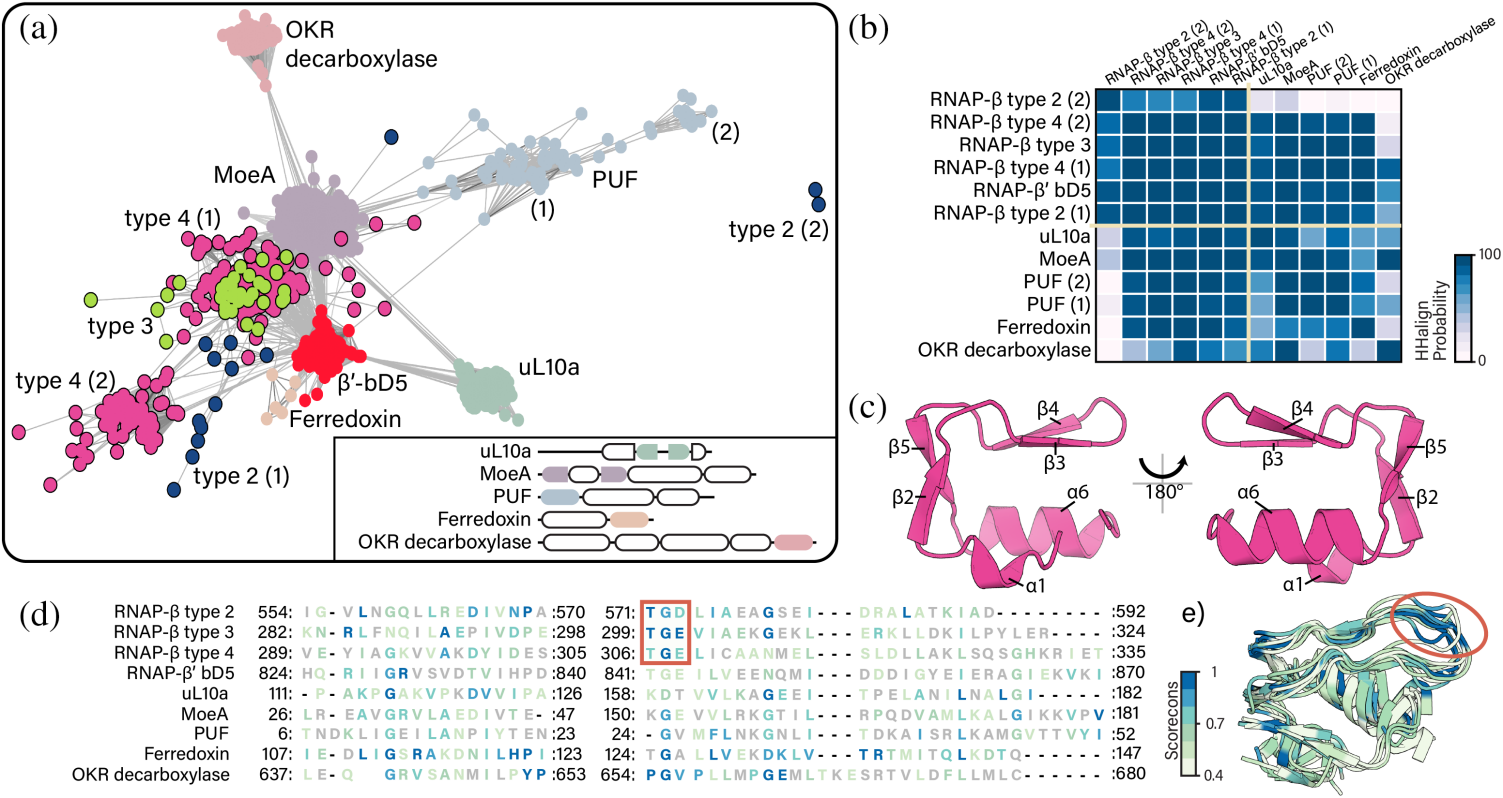
The BEAN domain. (a) BEAN domain sequences clustered by similarity at a P‐value threshold of 1 × 10^−12^. Inset: Multi‐domain organization of representative proteins containing BEAN domains. (b) Similarity matrix of pairwise comparison of full‐length BEAN domain HHalign profiles. High HHalign probability (>70%) suggests homology. (c) Structure of the BEAN domain (AlphaFold DB: AF‐P0A8V4‐F1). (d) Structure‐derived multiple sequence alignment of type 2 RNAP‐β; type 3 RNAP‐β; type 4 RNAP‐β; type 1 RNAP‐β′; uL10a; MoeA; a hypothetical protein; ferredoxin; and ornithine/lysine/arginine decarboxylase. (e) Structural superimposition of the same proteins. Source of three dimensional structures: type 2 RNAP‐β (AlphaFold DB: AF‐A9B6J3‐F1); type 3 RNAP‐β (AlphaFold DB: AF‐Q8ETY8‐F1); type 4 RNAP‐β (AlphaFold DB: AF‐P0A8V4‐F1); type 1 RNAP‐β′ (AlphaFold DB: AF‐Q0AUH3‐F1); uL10a (AlphaFold DB: AF‐Q8TZJ8‐F1); MoeA (AlphaFold DB: AF‐O59354‐F1); a hypothetical protein (AlphaFold DB: AF‐B8J6M3‐F1); ferredoxin (AlphaFold DB: AF‐Q4C556‐F1); and ornithine/lysine/arginine decarboxylase (AlphaFold DB: AF‐P52095‐F1). The structure‐derived multiple sequence alignment and the structural superimposition are colored by Scorecons. Residues with scores below 0.4 are gray.

RNAP‐β contains b/lineage‐specific domains at multiple positions (Figures 1a and S1a). These idiosyncratic domains are inserted at five distinct sites of RNAP‐β: in the (i) N‐terminal half of β‐uD2; (ii) C‐terminal half of β‐uD2; (iii) N‐terminal half of β‐uD3; (iv) C‐terminal half of β‐uD5; and (v) C‐terminal half of β‐uD4. The b‐ and b/lineage‐specific domains in RNAP‐β are less conserved in sequence than universal domains (Figure 1c–f). Most b/lineage‐specific insertional domains share structure (Figure 1g) and sequence similarity (Figure 3a,b), suggesting common ancestry. We call these domains BEAN (broadly embedded annex). To locate the boundaries of BEAN domains and isolate them for further analysis, we used the MSA block structure as well as CATH domain assignments. The BEAN domain maps to CATH superfamily 3.90.105.10.

Sites of BEAN insertion within RNAP‐β define b/lineage‐specific RNAP‐β architecture (Figure 1a). We distinguish four RNAP‐β types (architectures). Type 1 RNAP‐β lacks b/lineage‐specific insertions (Figure 1a,c). Type 2 RNAP‐β has a BEAN domain inserted within β‐uD3 (Figure 1a,d). Type 3 RNAP‐β has a BEAN domain inserted within β‐uD2 (Figure 1a,e). Type 4 RNAP‐β has a BEAN domain inserted within β‐uD2 (Figure 1a,f). Certain RNAP‐β proteins display a type 4 architecture with additional multidomain insertions; these are referred to as type 4*.

### 2.3 The BEAN domain

The core of the BEAN domain has a characteristic three‐dimensional structure composed of two square bracket‐like elements that are anti‐parallel relative to each other (Figure 3c). Each bracket‐like element is formed by an α‐helix and two β‐strands. The relative orientation of consecutive secondary elements within each bracket is 90°. The first bracket is formed by α1⊥β2⊥β3 and the second bracket by β4⊥β5⊥α6. Some BEAN domains are elaborated by insertions of additional secondary structural elements.

We identify BEAN domains in bacterial and archaeal proteins other than RNAP‐β (Figure 3). BEAN domain sequences in RNAP‐β and other proteins were isolated and compared all against all. Our characterization of BEAN as a homologous domain is based on full‐domain sequence similarity (BLAST+ *p*‐value <1 × 10^−12^ and HHalign probability >70%, Figure 3a,b). We find BEAN domains in: bacterial RNAP‐β′; the archaeal version of ribosomal protein uL10 but not in bacterial uL10; molybdenum cofactor biosynthesis protein MoeA; ornithine/lysine/arginine (OKR) decarboxylases; a putative ferredoxin; and in one protein of unknown function (PUF). In archaeal uL10, the BEAN domain is insertional and hosted by the core uL10 domain (Figures 3a and S4). In MoeA and PUF, BEAN is a N‐terminal appendix. In ferredoxin and OKR decarboxylases BEAN is a C‐terminal appendix.

Sequence similarities based on BLASTP P‐values show that b/lineage‐specific BEAN domains in RNAP‐β are more similar to each other than to BEAN domains in other proteins (Figure 3a). Additionally, BEAN domains in RNAP‐β show a conserved TGD/E sequence motif (threonine, glycine, aspartic/glutamic acid, Figure 3d) that is absent from other BEAN domains. Thus, BEAN domains in RNAP‐β appear to share more recent ancestry with each other than with BEAN domains of other proteins. However, due to their small size and high sequence divergence (Figure 3d,e), a statistically supported phylogenetic reconstruction of BEAN domains could not be calculated.

### 2.4 RNAP‐β′ multi‐domain architectures

RNAP‐β′ is encoded by two genes in archaea (rpo1N and rpo1C). Rpo1N and Rpo1C assemble to form a complete RNAP‐β′, which is also called Rpo1, in archaea (Korkhin et al., 2009). Our naming system for domains in archaeal RNAP‐β′ follows the continuous order from Rpo1N to Rpo1C. Domains β′‐uD1 to β′‐uD4 are common to bacterial RNAP‐β′ and archaeal Rpo1N; and domains β′‐uD5 and β′‐uD6 are common to bacterial RNAP‐β′ and archaeal Rpo1C (Figures 2a and S3e). The DΨBB domain of RNAP‐β′ is β′‐uD2.

We identified a‐specific insertional domains within β′‐uD2 and β′‐uD3 (Figures 2a and S3e). We also identified three a‐specific domains in Rpo1C: β′‐aD3, β′‐aD4 and β′‐aD5. β′‐aD3 is an N‐terminal addition to Rpo1C; β′‐aD4 is inserted into β′‐uD5, and β′‐aD5 is inserted into β′‐aD4.

**FIGURE 4:**
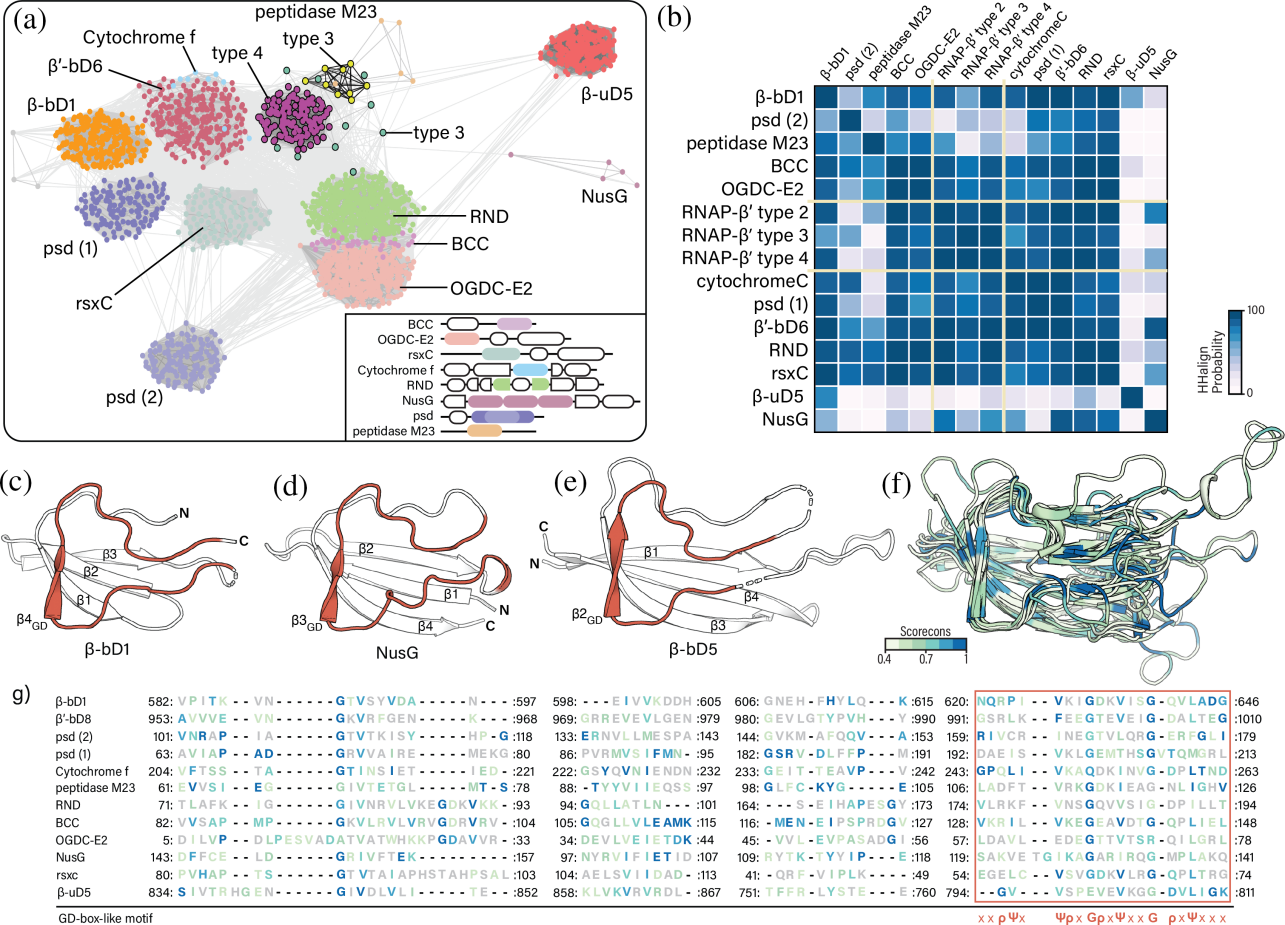
The HABAS domain. (a) HABAS domains clustered by sequences similarity at a *p*‐value threshold of 1 × 10^−9^. Inset: Multi‐domain organization of representative proteins containing HABAS domains. (b) Sequence similarity matrix based on pairwise comparison of full‐length HABAS domain profiles. Higher HHalign probability (>70%) suggests homology. (c–e) Structure representation of HABAS domains. The GD‐box‐like motif is highlighted in dark orange. (c) HABAS domain β4_GD_ topology (AF‐A2BPU4‐F1). Residues 616–625 are masked. (d) HABAS domain β3_GD_ topology (AF‐P77611‐F1). (e) HABAS domain β2_GD_ topology (AF‐C5CGE4‐F1). Residues 711–725 and 747–775 are masked. Structure‐derived multiple sequence alignment (f) and structure superimposition (g) of β‐bD1 (A2BT61), β′‐D8 (Q0AUH3), psd (A0L627), cytochrome f (A2BPU4), peptidase M23 (A0A0E3QUJ6), RND (A0A1T5M7J5), BCC (O59021), OGDC‐E2 (P0AFG6), NusG (C5CGE4), rsxC (P77611) and β‐uD5 (P11512). The structure‐derived multiple sequence alignment and the structure superimposition are colored by Scorecons conservation. Residues below 0.4 are gray.

Bacterial RNAP‐β′ is composed of six universal RNAP‐β′ domains and seven b‐specific domains (type 1, Figure 2a). Domains β′‐bD1, β′‐bD2, β′‐bD3, β′‐bD4, β′‐bD6 and β′‐bD7 are insertional. β′‐bD5 is a BEAN domain and β′‐bD6 is a HABAS domain. β′‐bD3 is inserted into the DΨBB domain of the β′ subunit and shows no sequence or structure similarity to the archaeal DΨBB domain insertion (β′‐aD1). β′‐bD2 and β′‐bD7 are homologous (CATH superfamily 3.30.60.280, Figure S2c,d). Here, we call these domains sLC (small left claw).

b/lineage‐specific insertions in RNAP‐β′ are observed in seven distinct locations (Figure 2a): (i) in the first half of β′‐bD2; (ii) in the middle of β′‐bD2; (iii) in the middle of β′‐bD4; (iv) in the second half of β′‐bD5; (v) near the N‐terminus of β′‐uD5; (vi) in the second half of β′‐bD6; and (vii) in the middle of β′‐bD7. Based on the presence and location of b/lineage‐specific insertions, we defined four main types of bacterial RNAP‐β′ (Figure 2a). Type 1 RNAP‐β′ in bacteria has no b/lineage‐specific insertions. Types 2–4 bacterial RNAP‐β′ have b/lineage‐specific insertional domains. Most of these b/lineage‐specific insertions are HABAS domains, which share structural similarity (Figures 2g) and sequence similarity (Figures 4a,b and S2).

The location and number of b/lineage‐specific insertions define the ‘type’ of RNAP‐β′. Bacterial type 1 RNAP‐β′ contains a single BEAN domain (β′‐bD5) and single HABAS domain (β′‐bD6). In addition to the domains found in bacterial type 1 RNAP‐β′, bacterial type 2 RNAP‐β′ contains a BEAN insertion within β′‐bD2 and sometimes a HABAS domain within β′‐bD6 (Figure 2a,c). Bacterial type 3 and 4 RNAP‐β′ have HABAS domain insertions within RNAP β′‐uD5 (Figures 2a and S2a). These insertions differ in number but not in location. For example, bacterial type 4 RNAP‐β′ contains two additional HABAS domains (Figure 2a,d) whereas bacterial type 3 RNAP‐β′ contains nine additional HABAS domains (Figure 2a,e). It appears that type 3 RNAP‐β′ is an elaboration of type 4 RNAP‐β′. HABAS domains in type 3 RNAP‐β′ are inserted into other HABAS domains. These recursively inserted domains in some cases exchange secondary structural elements (are domain swapped), forming a complex interdigitated structure (Figure 2e) called Si3 (Chlenov et al., 2005; Qayyum et al., 2024). Beyond the well‐populated types described here, 11 additional RNAP‐β′ variants are observed, with various combinations of HABAS, BEAN and sLC domains inserted within β′‐bD2, β′‐bD4, β′‐bD5 and β′‐bD7. This collection includes RNAP‐β′ architectures with one or a few (three or less) representatives in our dataset of species (Table S4). All these species are in deeply rooted lineages such as Firmicutes and the DST group (deinococcus‐thermus, synergistetes, thermotogae and related bacteria).

Note that bacterial type 3 RNAP‐β′ is composed of two polypeptide chains. The N‐terminal sub‐subunit (RNAP‐β′_BacN_) ends with β′‐uD3; and the C‐terminal sub‐subunit (RNAP‐β′_BacC_) starts at β′uD4 (Figure 2a). RNAP‐β′_BacN_ and RNAP‐β′_BacC_ assemble to form a complete RNAP‐β′ (Qayyum et al., 2024).

### 2.5 The HABAS domain

Many b/lineage‐specific insertions in RNAP subunit β*′* are HABAS domains (CATH superfamily 2.40.50.100). The HABAS domain is also observed as universal domain in RNAP‐β (β‐uD5), and as a b‐specific domain in RNAP‐β (β‐bD1). Full‐domain sequence comparisons reveal additional proteins containing HABAS domains (BLAST+ *p*‐value <1 × 10^−9^ and HHalign probability >70%, Figure 4a,b). Proteins with HABAS insertions are involved in metabolism (phosphatidylserine decarboxylase proenzyme, biotin carboxyl carrier protein, ion‐translocating oxidoreductase complex subunit C, cytochrome f), transport (major facilitator superfamily transporter protein, resistance‐nodulation‐division family transporter protein) and genetic information processing (transcription termination/antitermination protein NusG) (Figure 4).

The HABAS domain is a four‐stranded open β‐sheet with a conserved sequence motif in one of the β‐strands and the adjoining loop. The conserved motif contains glycine (G), aliphatic (Ψ), and polar (ρ) amino acids as follows: ΨxΨρxGρxΨxxGρxΨxx. We call this motif the GD‐box‐like motif because it is similar but not identical to the GD‐box sequence motif ΨxΨxxGρxΨxΨ (Alva et al., 2009). We found three distinct topologies of secondary structural elements in HABAS domains (Figure 1d–f). These topologies are related by circular permutation. We distinguish and name these topological variants by the locations of their GD‐box‐like motifs. The most frequently observed topology has a GD‐box‐like motif in strand β4 (β4_GD_), we refer to it as a β4_GD_ topology (Figure 4c). NusG has a β3_GD_ topology (Figure 4d), and a HABAS domain of β‐uD5 has a β2_GD_ topology (Figure 4e).

### 2.6 Phylogenetic distribution of RNAP‐β and RNAP‐β′ types

**FIGURE 5:**
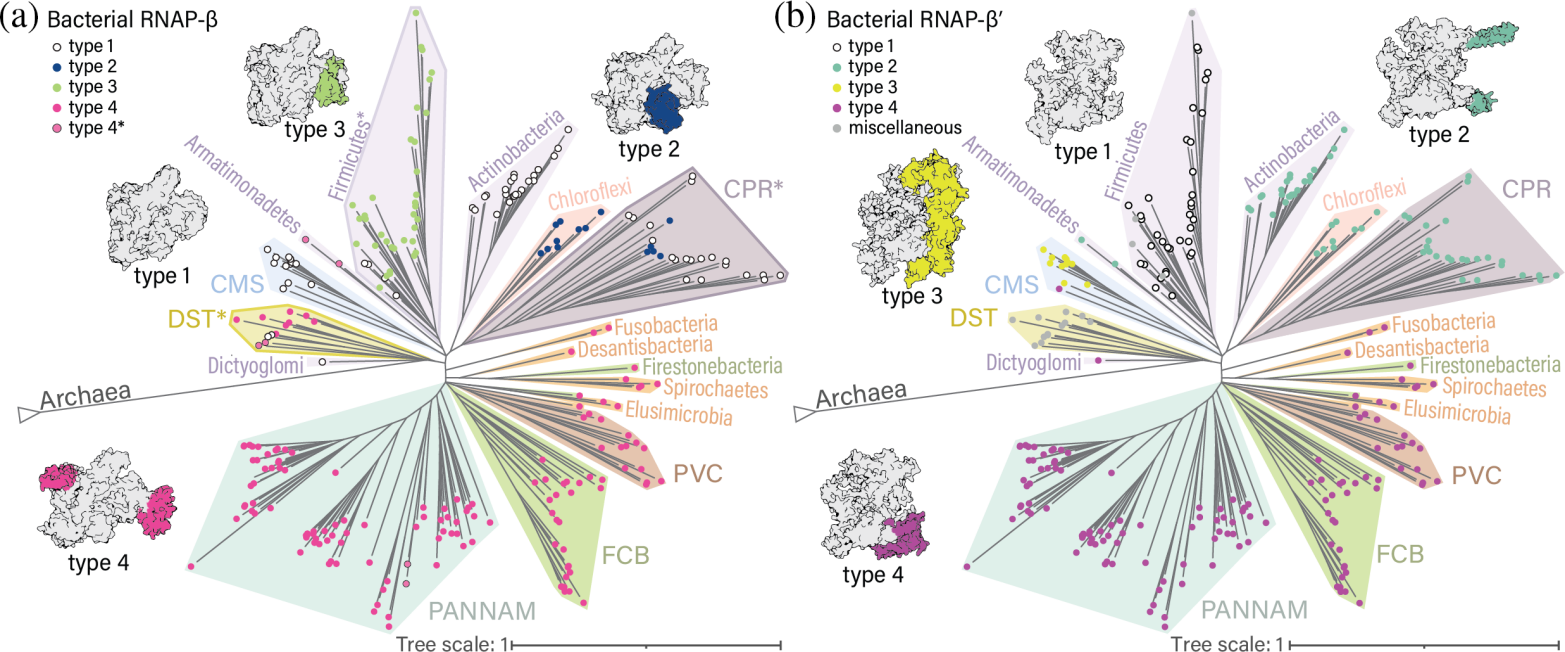
Phylogenetic distribution of various domain organizations of RNAP‐β and RNAP‐β′ in bacteria. Domain organization types are indicated by color. The tree of bacteria was adapted from Moody et al. (2022). (a) Distribution of RNAP‐β types in bacteria. Phylogenetic groups with a scattered distribution of type 1 RNAP‐β are indicated by a darker outline. (b) Distribution of RNAP‐β types in bacteria. CMS: Cyanobacteria, Margulisbacteria, Melainabacteria; CPR: Candidatus Phyla Radiation; DST: Deinococcus‐Thermus, Synergistes, Thermotogae, Bipolaricaulota, Caldiserica, Coprothermobacterota; FCB: Fibrobacteres, Chlorobi, Bacteroides, Gemmatimonadetes, Candidatus Cloacimonetes, division KSB1, Eisenbacteria, Candidatus Fermentibacteria, Firestonebacteria, Candidatus Glassbacteria, Ignavibacteria, Kryptonia, Marinimicrobia, Raymondbacteria, Stahlbacteria, Zixibacteria; PANNAM: Bdellovibrio, Dependentia, Proteobacteria, Aquificae, Myxococcota, Nitrospinae, Nitrospirae, Acidobacteria, Chrysiogenetes, Deferribacteres, Schekmanbacteria and Thermodesulfobacteria; PVC: Planctomycetes, Verrucomicrobia, Chlamydiae, Kiritimatiellaeota, Lentisphaerae, Candidatus Desantisbacteria, Candidatus Omnitrophica.

The phylogenetic distribution of RNAP‐β and RNAP‐β′ types as we define them here follows the deeply rooted divergence of Gracilicutes and Terrabacteria (Coleman et al., 2021; Witwinowski et al., 2022). We identified type 4 RNAP‐β and type 4 RNAP‐β′ in most Gracilicutes; by contrast, we identified all RNAP‐β and RNAP‐β′ types in Terrabacteria (Figure 5). Type 4 RNAP‐β appears characteristic of Gracilicutes but is also observed in DST and Armatimonadetes.

A given bacterial lineage tends to have a single type of RNAP‐β. Bacteria in the CMS group (Cyanobacteria and related bacteria) contain type 1 RNAP‐β; Armatimonadetes contain type 4* RNAP‐β; Actinobacteria contain type 1 RNAP‐β; and Chloroflexi contain type 2 RNAP‐β. However, type 1 is scattered among other types of RNAP‐β in some bacterial lineages. Most bacteria in the DST group contain type 4 RNAP‐β, but some contain type 1. Firmicutes generally contain type 3 RNAP‐β, but some contain type 1. Bacteria in the CPR group (candidate phyla radiation) contain either type 2 or type 1 RNAP‐β.

**FIGURE 6:**
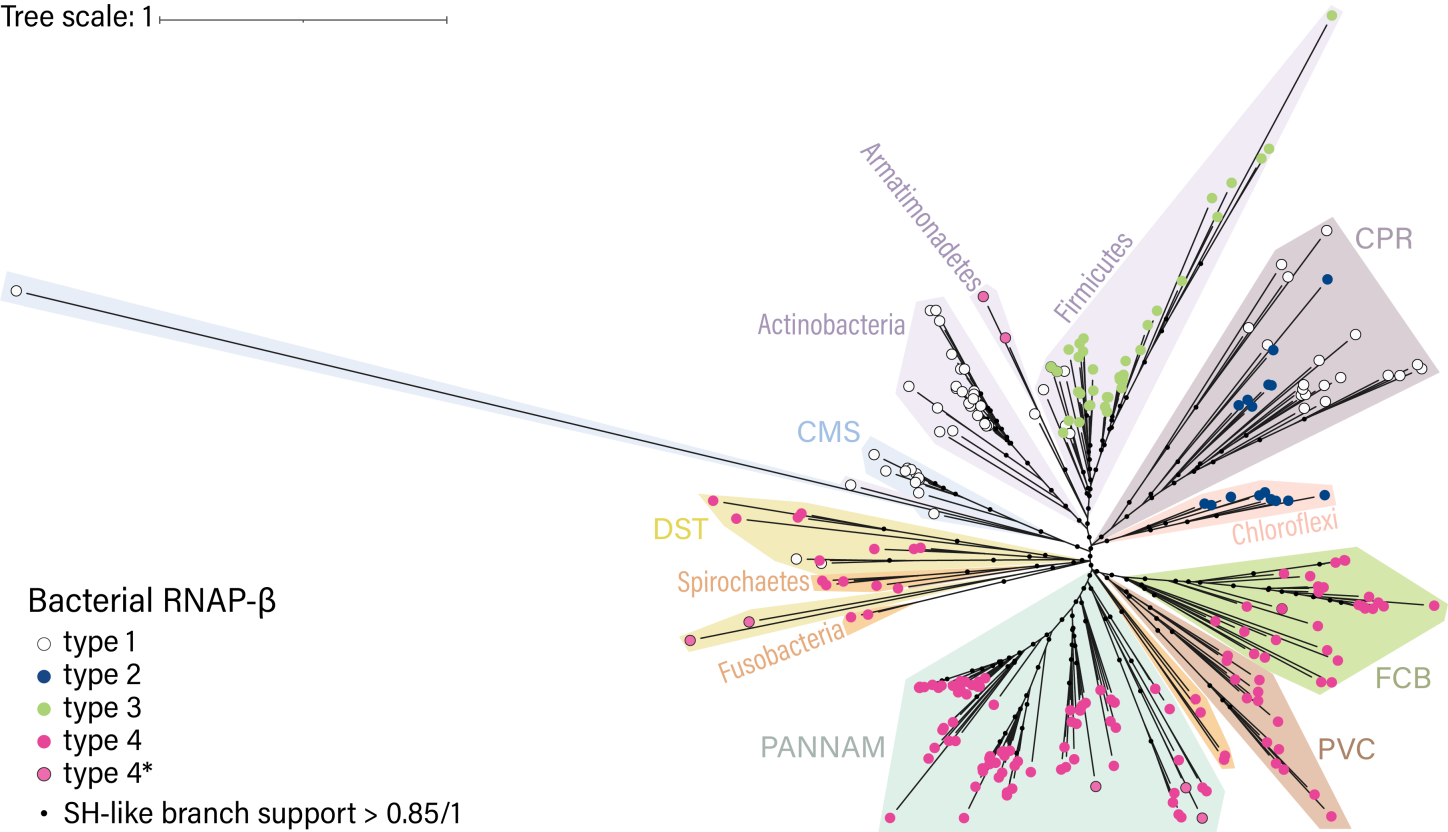
RNAP‐β maximum likelihood tree. The maximum likelihood tree of RNAP‐β was calculated using positions conserved in all bacteria. The MSA of RNAP‐β was trimmed with TrimAl to remove highly gapped positions (the gap threshold of 0.9 removed positions with gaps in more than 10% of sequences).

Type 1 RNAP‐β lacks b/lineage‐specific BEAN insertions. The scattered phylogenetic distribution of Type 1 RNAP‐β could result from HGT or from reduction from more elaborate types. To test whether the scattered distribution of type 1 RNAP can be attributed to HGT, we calculated a maximum likelihood gene tree of RNAP‐β using sites that are conserved in all bacteria (Figure 6). We compared the gene tree of RNAP‐β to a consensus tree of bacteria calculated previously using 27 vertically inherited genes (Moody et al., 2022). Our phylogenetic analysis shows that the sequences of RNAP‐β group by species (Figures 6) and suggests vertical inheritance of the RNAP‐β gene in DST, Firmicutes and CPR. This correspondence further suggests that type 1 in DST, Firmicutes and CPR evolved by reduction through loss of b/lineage‐specific BEAN insertions.

## 3 DISCUSSION

**FIGURE 7:**
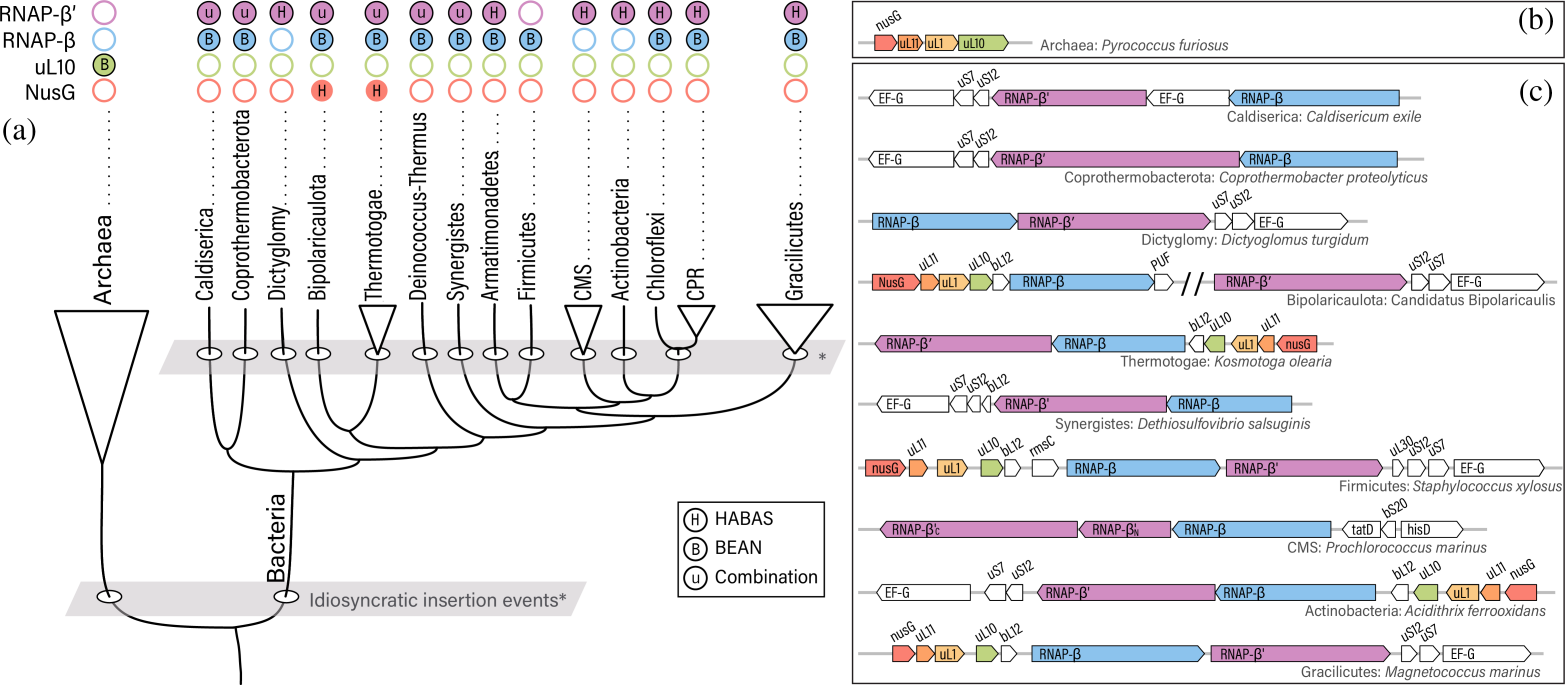
RNAP‐β and RNAP‐β′ domain insertions mapped into a schematic representation of the tree of bacteria. The tree reproduces the topology from Figure 5a,b, branches lengths have been altered. (a) RNAP‐β′ with unique combinations of BEAN, HABAS sLC insertions. (b) Most frequent genome contexts for uL10 in archaea (Table S3). (c) Most frequent genome context for RNAP‐β and RNAP‐β′ in bacteria (Tables S1 and S2).

The data presented here are consistent with a model in which RNAP was subject to a discrete episode of aggressive domain insertion, around or after the last bacterial common ancestor, followed by a precipitous decline in the frequency of insertion (Figure 7). RNAP is a multi‐subunit protein complex that contains RNAP‐β and RNAP‐β′ subunits. RNAP‐β and RNAP‐β′ are found in RNAPs in archaea, bacteria, eukarya, and nucleocytoplasmic large DNA viruses (Iyer et al., 2001). Here we report that RNAP‐β and RNAP‐β′ each contain homologous insertional domains with idiosyncratic positions that generate block structures of RNAP‐β and ‐β′ MSAs. The locations and phylogenetic distributions of insertional domains in RNAP‐β, RNAP‐β′ and uL10 report on events that occurred in the deep evolutionary past. These insertional domains appear in distinct positions in the most deeply rooted bacterial lineages.

Block structures of MSAs are not exclusive to RNAP‐β and RNAP‐β′ and have been described for universal components of the translation system [ribosomal proteins (Vishwanath et al., 2004), and aminoacyl tRNA synthetases (Alvarez‐Carreño et al., 2023)]. But block differences in the translation system are observed between archaea and bacteria whereas here, in RNAP, they are observed within archaeal and bacterial domains. The presence of homologous insertional domains in idiosyncratic positions pose important questions about evolutionary mechanisms.

We call the most common RNAP‐β and RNAP‐β′ insertional domains BEAN and HABAS. The BEAN domain has a characteristic three‐dimensional structure composed of two square bracket‐like elements that are antiparallel relative to each other. Each bracket‐like element is formed by an α‐helix and two β‐strands (Figure 3c). The orientation between consecutive secondary elements is 90° within each bracket. The HABAS domain contains a four‐stranded open β‐sheet with a GD‐box‐like motif in one of the β‐strands and the adjoining loop (Figure 4 c‐e). In some instances, recursively inserted HABAS domains form complex domain‐swapped structures.

### 3.1 b/lineage‐specific HABAS and BEAN domain insertions are polyphyletic

Insertional domains interrupt universal domains of RNAP‐β and RNAP‐β′, and thus, post‐date the establishment of the basic multi‐domain architectures of RNAP‐β and RNAP‐β′. Insertions occur in distinct locations, allowing us to establish ‘types’ of RNAP‐β and RNAP‐β′. BEAN insertions specify the type of RNAP‐β. HABAS domains specify the type of RNAP‐β′. We clustered RNAP‐β and RNAP‐β′ within bacteria based on number and kind of insertional domains. The types of RNAP‐β and RNAP‐β′ follow the tree of bacteria. BEAN and HABAS domain are identified in a small number of defined locations in RNAP‐β and RNAP‐β′, which suggests that insertion of these domains occurred only a few times during evolution.

*BEAN insertions and RNAP‐β evolution*. The distribution of RNAP‐β types suggests that BEAN domains were independently inserted (are polyphyletic) in the ancestors of three early branching bacterial lineages (Figure 7). These lineages are: (i) the ancestor of Firmicutes, which acquired type 2 insertions; (ii) the ancestor of Chloroflexi and CPR bacteria, which acquired type 3 insertions; and (iii) the ancestor of Gracilicutes, which acquired type 4 insertions. Armatimonadetes and DST also have type 4 insertions. Type 4 RNAP‐β insertions could have arisen from HGT from Gracilicutes. It is also possible that BEAN insertion occurred in the ancestors of Armaitimonadetes, DST and Gracilicutes at the same location in the RNAP‐β gene. Thus, the location of these BEAN insertions would be convergent. Similarities in BEAN locations suggest that common characteristics of the genome context (Figure 7) could have influenced the insertion sites.

*HABAS insertions and RNAP‐β*′*evolution*. HABAS insertions in bacterial RNAP‐β′ appear to have occurred independently in three ancestral populations: (i) the ancestor of Armatimonadetes, Actinobacteria, Chloroflexi and CPR bacteria; (ii) an ancestor within the CMS group; and (iii) the ancestor of Gracilicutes. Extensive insertional diversity with the DST group suggests that these insertions occurred very early in bacterial evolution. The lack of insertional diversity in RNAP in late divergent groups suggests cessation of insertions in later bacterial evolution. Interestingly, predicted and experimentally determined structures of the chloroplast RNAP in *Sinapis alba* (do Prado et al., 2024) contain unique HABAS insertions in addition to type 3 RNAP‐β′ insertions (Figure S5). This observation provides additional information to relatively date HABAS insertions prior to the primary endosymbiosis of chloroplasts. The genes for RNAP‐β and RNAP‐β′ recorded and preserved the marks of evolutionary events that affected ancestral groups.

Acquisition of BEAN domains may have been independent of acquisition of HABAS domains. We observe a mismatch in the distributions of RNAP‐β and RNAP‐β′ (Figures 5 and 7). For example: (i) type 2 RNAP‐β assembles with type 1 RNAP‐β′ in CMS and Actinobacteria, or with type 2 RNAP‐β′ in Chloroflexi and CPR, or with type 4 RNAP‐β′ in Armatimonadetes; (ii) type 4 RNAP‐β assembles with type 1 RNAP‐β′ in Dictioglomy, or with type 4 RNAP‐β′ in Gracilicutes.

DST bacteria appear to be unique: they contain type 4 BEAN insertions in RNAP‐β, which are characteristic of Gracilicutes, as well as insertions in RNAP‐β′ that fall outside of our classification. Thus, RNAP‐β sequences from the DST group resemble Gracilicutes sequences, and DST RNAP‐β′ insertions are not observed elsewhere in Gracilicutes or Terrabacteria. In recent deep phylogenetic analysis, the placement of the DST group within Terrabacteria is unresolved (Moody et al., 2024). In our gene tree, the DST appear as a sister lineage of Gracilicutes. The topology of our tree does not allow us to rule out or support a HGT of type 4 RNAP‐β from Gracilicutes to DST.

The mechanisms of generation of these insertional idiosyncrasies remains unclear. One possibility is that HABAS and BEAN insertions were acquired all at once by different bacterial groups, and differences in location would reflect genomic differences (Figure 7). A second possibility is that insertion events were recurring, and different locations correspond to different episodes of insertion. Another possibility is that BEAN and HABAS domains have shifted between locations in some bacterial lineages. Insertional domains in RNAP‐β, RNAP‐β′ and uL10a reveal a processes that reshaped the multidomain architecture of bacterial and archaeal orthologs and tapered off after early evolution.

### 3.2 Insertional domains in the evolution of translation and transcription

Sequence similarity searches indicate that HABAS and BEAN are inserted in multiple unrelated proteins. We identify a b‐specific BEAN insertion in RNAP‐β′ and b/lineage specific BEAN insertions in bacterial RNAP‐β. We observe BEAN insertions in the archaeal version of ribosomal protein uL10. We identify a universal HABAS insertion and a b‐specific insertion of HABAS in RNAP‐β, and b/lineage specific HABAS insertions in RNAP‐β′. Finally, we observe HABAS insertions in NusG, the only universally conserved transcription elongation factor (Werner & Grohmann, 2011). HABAS insertions in NusG were identified only in bacteria from the DST group: *Fervidobacterium islandicum*, *Petrotoga olearia*, *Kosmotoga olearia* and *Candidatus* Bipolaricaulis anaerobius (Table S2). The observation of a BEAN domain in the archaeal but not in the bacterial version of universal ribosomal protein L10 suggests insertion after the last universal common ancestor (LUCA).

The local gene neighborhood may have influenced the acquisition of BEAN and HABAS domains. The genes for RNAP‐β and ‐β′ are adjacent to each other in the genomes of virtually all bacteria and most are in the neighborhood of the genes that encode for NusG and universal ribosomal proteins uL1 and uL11 (Tables S2 and S2). Similarly, in most archaea, uL10 is in the neighborhood of the genes that encode for NusG, uL1 and uL11 (Table S3).

Transcription and translation are the central biological processes responsible for the encoding and synthesis of proteins. The patterns of insertion of HABAS and BEAN domains in universal and ancient proteins pose provocative questions regarding the timing and order of events during the early evolution of life. The mechanism of insertion remains unclear.

The combined data suggest that the bulk of the acquisition of BEAN domains in RNAP‐β and archaeal uL10 and HABAS domains in RNAP‐β′ occurred in ancestral lineages, shortly after LUCA, and that the descendants generally retained these insertions. We speculate that BEAN and HABAS insertions could have been influenced by the genomic context. The slight differences in the locations of BEAN and HABAS insertions in RNAP‐β and RNAP‐β′ may reflect distinct bacterial lineages with distinct gene locations. Thus, in our model, b/lineage specific occurred in the deep evolutionary past, and just after an early divergence of the Last Bacterial Common Ancestor into distinct bacterial groups. The patterns that we observe left a mark on some of the first ancestral bacterial groups, and hint to an early diversification of Terrabacteria, particularly of the DST group.

## 4 METHODS

### 4.1 Identification of RNAP‐β and β′ subunits in bacteria and archaea

The sequences of RNAP subunits β and β′ from *Sulfolobus acidocaldarius* (UniProt IDs: P11513 and P11512) and *Bacillus subtitlis* (UniProt IDs: P37870 and P37871) were searched in a set of archaeal and bacterial proteomes derived from Moody et al. (2022) using phmmer from the HMMER3 suite (Eddy, 2011). Sequences below threshold (*E*‐value <1 × 10^−10^) were retrieved and aligned. Multiple sequence alignments (MSAs) were generated with the einsi option from MAFFT v7 (Katoh & Standley, 2013). The genome context of RNAP‐β and ‐β′ homologs were retrieved from NCBI and visualized with gggenes (Wilkins, 2020).

### 4.2 Domain annotation of RNAP‐β and β′ and classification

The MSAs of RNAP subunits β and β′ were converted each into a sequence profile and compared to CATH_S40, ECOD_F70 and SCOPe95 with HH‐search (Steinegger, Meier, et al., 2019) on the MPI Bioinformatics Toolkit (Zimmermann et al., 2018). CATH_S40 contains CATH domains clustered at 40% sequence identity; ECOD_F70 contains ECOD domains clustered at 70% sequence identity; and SCOPe95 contains domain sequences clustered at 95% sequence identity. The block patterns on the MSAs were used as reference to classify the multi‐domain organization types of bacterial RNAP‐β and RNAP‐β′ proteins (Figures S1 and S2). For each RNAP‐β and RNAP‐β′ type, a representative was selected for structure analysis (Tables 1 and 2). All representatives have a experimentally determined structure in the PDB (Berman et al., 2000) or a predicted structure in AlphaFold DB (Varadi et al., 2022). Per‐residue confidence score (pLDDT) and predicted aligned error (PAE) of the structure predictions are shown in Figure S6. Sequences with unique insertion patterns were annotated individually, and the annotations were inspected over structure predictions generated with AlphaFold version 2.0 (Jumper et al., 2021).

### 4.3 Identification of HABAS and BEAN domains homologs

A‐, b‐ and b/lineage‐specific insertions were trimmed according to the blocks in the MSAs. Profiles were calculated for each trimmed MSA with hhmake from the hh‐suite (Steinegger, Meier, et al., 2019), considering columns with fewer than 50% gaps match states.

The MSAs were converted to profile Hidden Markov Models using the HH‐suite version 3.3.0 (Steinegger, Meier, et al., 2019). The profiles were searched against the BFD database (Steinegger, Mirdita, et al., 2019) of clustered genome and metagenome sequences using HHblits (three iterations, probability >60). Significant matches (minimum probability: 60, minimum coverage with master sequence 80%) were retrieved and clustered with CLANS (Gabler et al., 2020) by all‐against‐all BLASTP sequence similarity (*p*‐value 1 × 10^−20^). Groups with at least 30 homologs in the cluster map were extracted; realigned with MAFFT einsi (Katoh & Standley, 2013); and converted to HMM profiles with HMMER version 3.3.2 (Eddy, 2011). The HMM profiles were searched with phmmer in the same set of archaeal and bacterial proteomes (Moody et al., 2022) that was used RNAP‐β and RNAP‐β′ identification.

Structure based MSAs of HABAS and BEAN domains were calculated with MATRAS (Kawabata, 2003). All‐against‐all structure comparisons of BEAN and HABAS domains were calculated with SSAP (Taylor & Orengo, 1989).

### 4.4 Maximum likelihood tree of RNAP‐β

The MSA of bacteria RNAP‐β was trimmed with trimAl v1.4 to remove positions with more than 10% gaps. The ML tree was calculated with PhyML (Guindon et al., 2010) on the Montpellier Bioinformatics Platform. Model selection was determined with SMS (Lefort et al., 2017). The ML tree was inferred with the Q.yeast +G + I model. Visualization of the tree was made with iToL (Letunic & Bork, 2021).

## AUTHOR CONTRIBUTIONS

**Claudia Alvarez‐Carreño:** Conceptualization; investigation; writing – original draft; writing – review and editing; formal analysis; data curation; methodology. **Angela T. Huynh:** Data curation; formal analysis. **Anton S. Petrov:** Writing – review and editing; formal analysis. **Christine Orengo:** Writing – review and editing. **Loren Dean Williams:** Writing – original draft; writing – review and editing; supervision.

## CONFLICT OF INTEREST STATEMENT

The authors have no conflicts of interest to declare that are relevant to the content of this article.

## Supporting information


**APPENDIX S1:** Supporting information.

**APPENDIX S2:** Supporting information.

## Data Availability

Sequence alignments and structure predictions associated with this manuscript have been deposited in the FigShare repository DOI: 10.6084/m9.figshare.25663923.
