# Supplementary material for: BEAN and HABAS: Polyphyletic insertions in the DNA‐directed RNA polymerase
Source: Protein Sci. 2024 Oct 28;33(11):e5194. doi: 10.1002/pro.5194 (PMC11515920; doi:10.1002/pro.5194)
Supplement: Supplementary file 1 — APPENDIX S1: Supporting information. [file PRO-33-e5194-s001.pdf]

Supplementary Materials

BEAN and HABAS: Polyphyletic insertions in the DNA-directed RNA polymerase

Claudia Alvarez-Carreño[1]*, Angela T. Huynh[2], Anton S. Petrov[2,3], Christine Orengo[1], Loren Dean Williams[2,3]*

[1]Department of Structural and Molecular Biology, University College London, London, United Kingdom

[2]School of Chemistry and Biochemistry, Georgia Institute of Technology, 901 Atlantic Dr, Atlanta, GA 30332, USA

[3]NASA Center for the Origin of Life, Georgia Institute of Technology, Atlanta, GA 30332-0400, USA

* Corresponding authors:

Claudia Alvarez-Carreño

Email: c.carreno@ucl.ac.uk

* Loren Dean Williams

Email: loren.williams@chemistry.gatech.edu

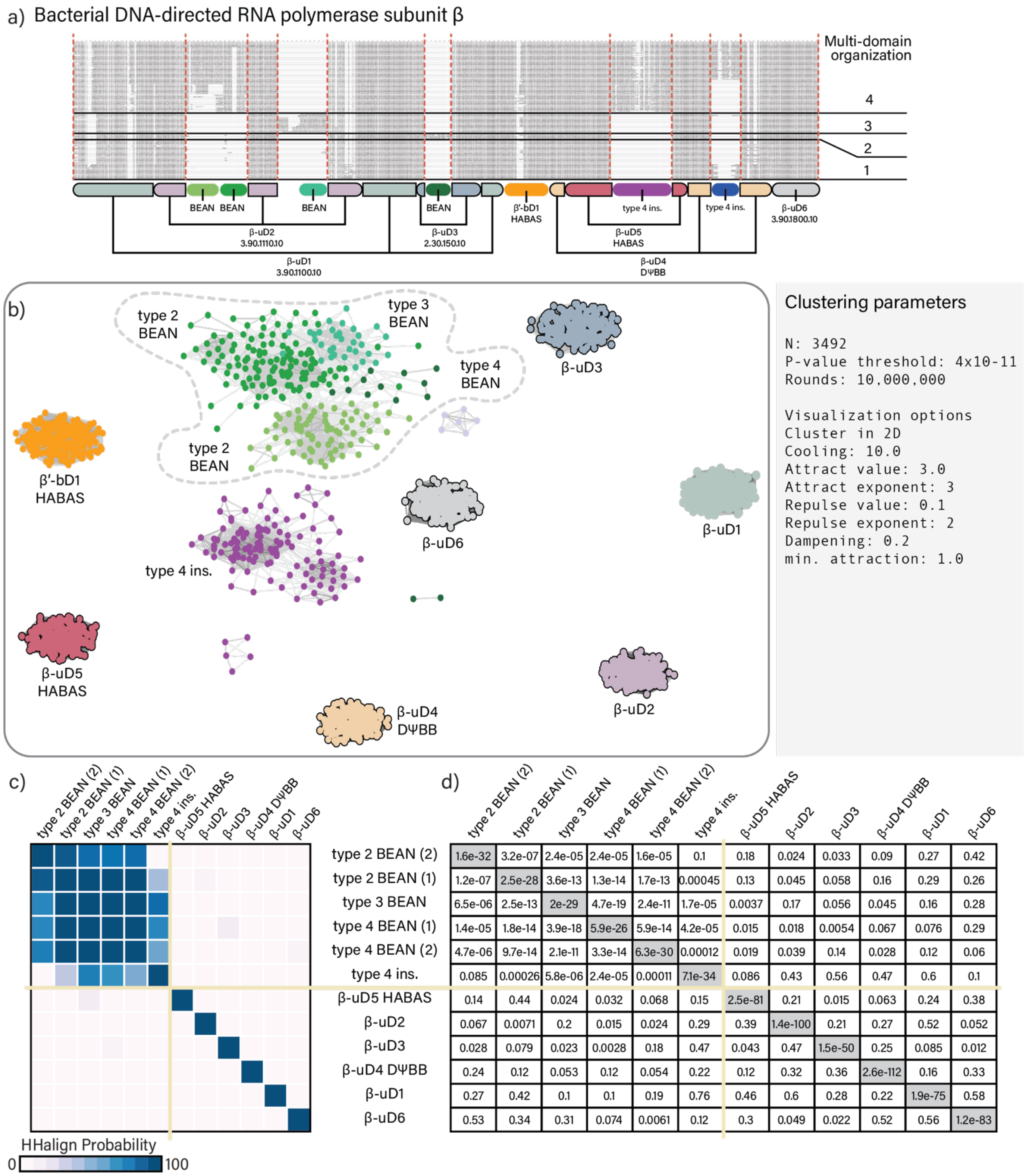

	type 2 BEAN (2)	type 2 BEAN (1)	type 3 BEAN	type 4 BEAN (1)	type 4 BEAN (2)	type 4 ins.	β-uD5 HABAS	β-uD2	β-uD3	β-uD4 DΨBB	β-uD1	β-uD6
type 2 BEAN (2)	1.6e-32	3.2e-07	2.4e-05	2.4e-05	1.6e-05	0.1	0.18	0.024	0.033	0.09	0.27	0.42
type 2 BEAN (1)	1.2e-07	2.5e-28	3.6e-13	1.3e-14	1.7e-13	0.00045	0.13	0.045	0.058	0.16	0.29	0.26
type 3 BEAN	6.5e-06	2.5e-13	2e-29	4.7e-19	2.4e-11	1.7e-05	0.0037	0.17	0.056	0.045	0.16	0.28
type 4 BEAN (1)	1.4e-05	1.8e-14	3.9e-18	5.9e-26	5.9e-14	4.2e-05	0.015	0.018	0.0054	0.067	0.076	0.29
type 4 BEAN (2)	4.7e-06	9.7e-14	2.1e-11	3.3e-14	6.3e-30	0.00012	0.019	0.039	0.14	0.028	0.12	0.06
type 4 ins.	0.085	0.00026	5.8e-06	2.4e-05	0.00011	7.1e-34	0.086	0.43	0.56	0.47	0.6	0.1
β-uD5 HABAS	0.14	0.44	0.024	0.032	0.068	0.15	2.5e-81	0.21	0.015	0.063	0.24	0.38
β-uD2	0.067	0.0071	0.2	0.015	0.024	0.29	0.39	1.4e-100	0.21	0.27	0.52	0.052
β-uD3	0.028	0.079	0.023	0.0028	0.18	0.47	0.043	0.47	1.5e-50	0.25	0.085	0.012
β-uD4 DΨBB	0.24	0.12	0.053	0.12	0.054	0.22	0.12	0.32	0.36	2.6e-112	0.16	0.33
β-uD1	0.27	0.42	0.1	0.1	0.19	0.76	0.46	0.6	0.28	0.22	1.9e-75	0.58
β-uD6	0.53	0.34	0.31	0.074	0.0061	0.12	0.3	0.049	0.022	0.52	0.56	1.2e-83

Figure S1. Blocks of conservation in the multiple sequence alignments of bacterial RNAP-β. (a) Multiple sequence alignment (MSA) of RNAP-β and domain annotation. Vertical lines delineate the blocks of differential conservation: universal; bacterial; and bacterial lineage specific. Horizontal lines delineate the different types of RNAP-β. Domain boundaries are indicated below the MSA. (b) Cluster of domain sequences based on pairwise BLAST+ P-values. Each dot represents a domain sequence. Dots are colored by domain as in (a). Connections between dots represent BLAST+ P-values below threshold ($4x10^{-11}$). The cluster was calculated with CLANS (2). (c-d) Similarity matrix showing pairwise profile-profile comparisons for each domain in RNAP-β. Profiles were calculated with hhmake (3) using the MSA in (a) trimmed to the domain boundaries. (c) HHalign probability scores shown as colours. (d) E-values of the pairwise comparisons.

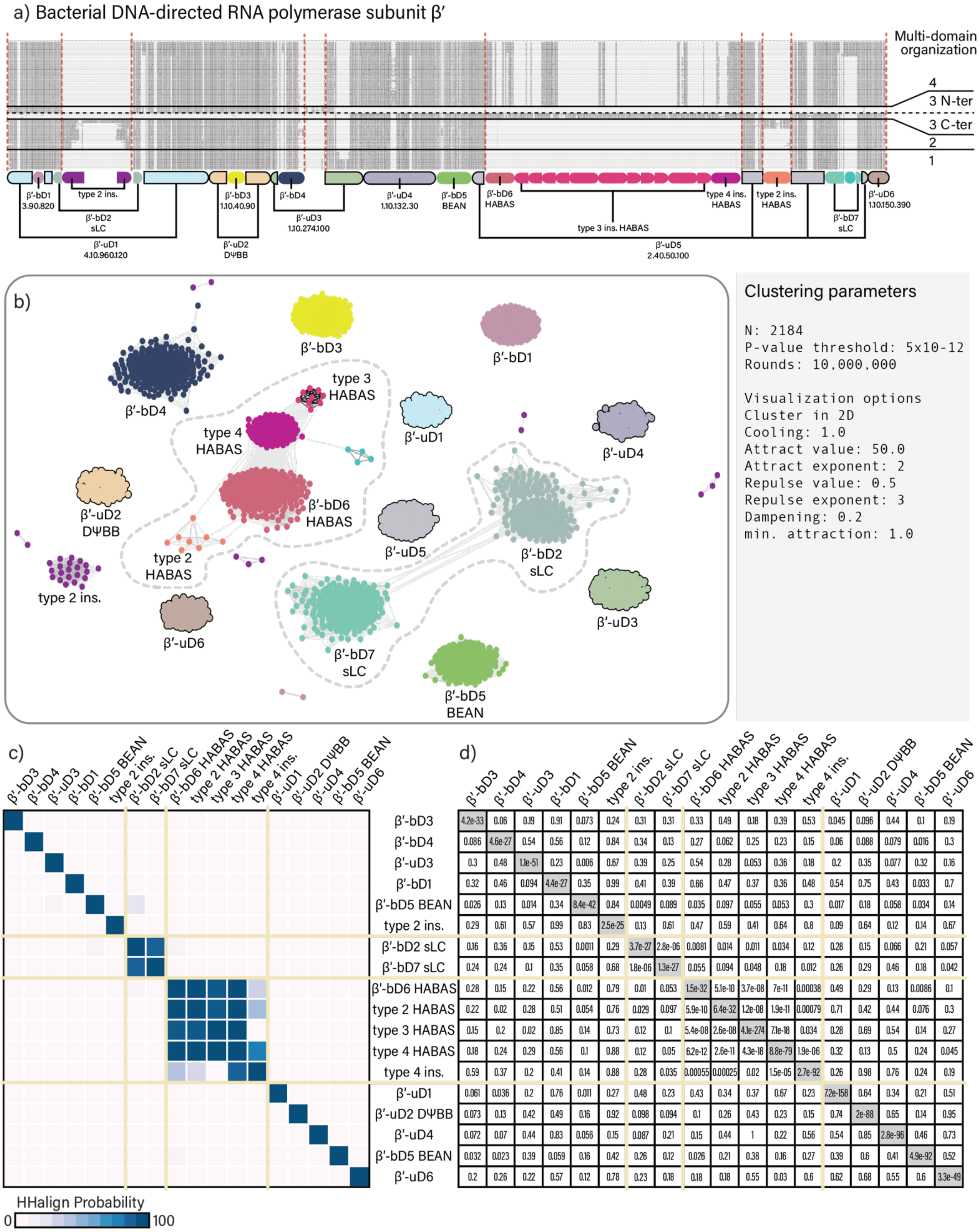

	β′-bD3	β′-bD4	β′-uD3	β′-bD1	β′-bD5 BEAN	type 2 ins.	β′-bD2 sLC	β′-bD7 sLC	β′-bD6 HABAS	type 2 HABAS	type 3 HABAS	type 4 HABAS	type 4 ins.	β′-uD1	β′-uD2 DΨBB	β′-uD4	β′-bD5 BEAN	β′-uD6
β′-bD3	4.2e-33	0.06	0.19	0.91	0.073	0.24	0.31	0.31	0.33	0.49	0.18	0.39	0.53	0.045	0.096	0.44	0.1	0.19
β′-bD4	0.086	4.6e-27	0.54	0.56	0.12	0.84	0.34	0.13	0.27	0.062	0.25	0.23	0.15	0.06	0.088	0.079	0.016	0.3
β′-uD3	0.3	0.48	1.1e-51	0.23	0.006	0.67	0.39	0.25	0.54	0.28	0.053	0.36	0.18	0.2	0.35	0.077	0.32	0.16
β′-bD1	0.32	0.46	0.094	4.4e-27	0.35	0.99	0.41	0.39	0.66	0.47	0.37	0.36	0.48	0.54	0.75	0.43	0.033	0.7
β′-bD5 BEAN	0.026	0.13	0.014	0.34	8.4e-42	0.84	0.0049	0.089	0.035	0.097	0.055	0.053	0.3	0.017	0.18	0.058	0.034	0.14
type 2 ins.	0.29	0.61	0.57	0.99	0.83	2.5e-25	0.13	0.61	0.47	0.59	0.41	0.64	0.8	0.09	0.64	0.12	0.14	0.67
β′-bD2 sLC	0.16	0.36	0.15	0.53	0.0011	0.29	3.7e-27	2.8e-06	0.0081	0.014	0.011	0.034	0.12	0.28	0.15	0.066	0.21	0.057
β′-bD7 sLC	0.24	0.24	0.1	0.35	0.058	0.68	1.8e-06	1.3e-27	0.055	0.094	0.048	0.18	0.012	0.26	0.29	0.46	0.18	0.042
β′-bD6 HABAS	0.28	0.15	0.22	0.56	0.012	0.79	0.01	0.053	1.5e-32	5.1e-10	3.7e-08	7e-11	0.00038	0.49	0.29	0.13	0.0086	0.1
type 2 HABAS	0.22	0.02	0.28	0.51	0.054	0.76	0.029	0.097	5.9e-10	6.4e-32	1.2e-08	1.9e-11	0.00079	0.71	0.42	0.44	0.076	0.3
type 3 HABAS	0.15	0.2	0.02	0.85	0.14	0.73	0.12	0.1	5.4e-08	2.6e-08	4.1e-274	7.1e-18	0.034	0.28	0.69	0.54	0.14	0.27
type 4 HABAS	0.18	0.24	0.29	0.56	0.1	0.88	0.12	0.05	6.2e-12	2.6e-11	4.3e-18	8.8e-79	1.9e-06	0.32	0.13	0.5	0.24	0.045
type 4 ins.	0.59	0.37	0.2	0.41	0.14	0.88	0.28	0.035	0.00055	0.00025	0.02	1.5e-05	2.7e-92	0.26	0.98	0.76	0.24	0.19
β′-uD1	0.061	0.036	0.2	0.76	0.011	0.27	0.48	0.23	0.43	0.34	0.37	0.67	0.23	7.2e-158	0.64	0.34	0.21	0.51
β′-uD2 DΨBB	0.073	0.13	0.42	0.49	0.16	0.92	0.098	0.094	0.1	0.26	0.43	0.23	0.15	0.74	2e-88	0.65	0.14	0.95
β′-uD4	0.072	0.07	0.44	0.83	0.056	0.15	0.087	0.21	0.15	0.44	1	0.22	0.56	0.54	0.85	2.8e-96	0.46	0.73
β′-bD5 BEAN	0.032	0.023	0.39	0.059	0.16	0.42	0.26	0.12	0.026	0.21	0.38	0.16	0.27	0.39	0.6	0.41	4.9e-92	0.52
β′-uD6	0.2	0.26	0.22	0.57	0.12	0.78	0.23	0.18	0.18	0.18	0.55	0.03	0.6	0.62	0.68	0.55	0.6	3.3e-49

Figure S2. Blocks of conservation in the multiple sequence alignments of bacterial RNAP-β′. (a) Multiple sequence alignment (MSA) of RNAP-β′ and domain annotation. Vertical lines delineate the blocks of differential conservation: universal; bacterial; and bacterial lineage specific. Horizontal lines delineate the different types of RNAP-β′. Domain boundaries are indicated below the MSA. (b) Cluster of domain sequences based on pairwise BLAST+ P-values. Each dot represents a domain sequence. Dots are colored by domain as in (a). Connections between dots represent BLAST+ P-values below threshold (5×10^{-12}). The cluster was calculated with CLANS (2). (c-d) Similarity matrix showing pairwise profile-profile comparisons for each domain in RNAP-β. Profiles were calculated with hhmake (3) using the MSA in (a) trimmed to the domain boundaries. (c) HHalign probability scores shown as colours. (d) E-values of the pairwise comparisons.

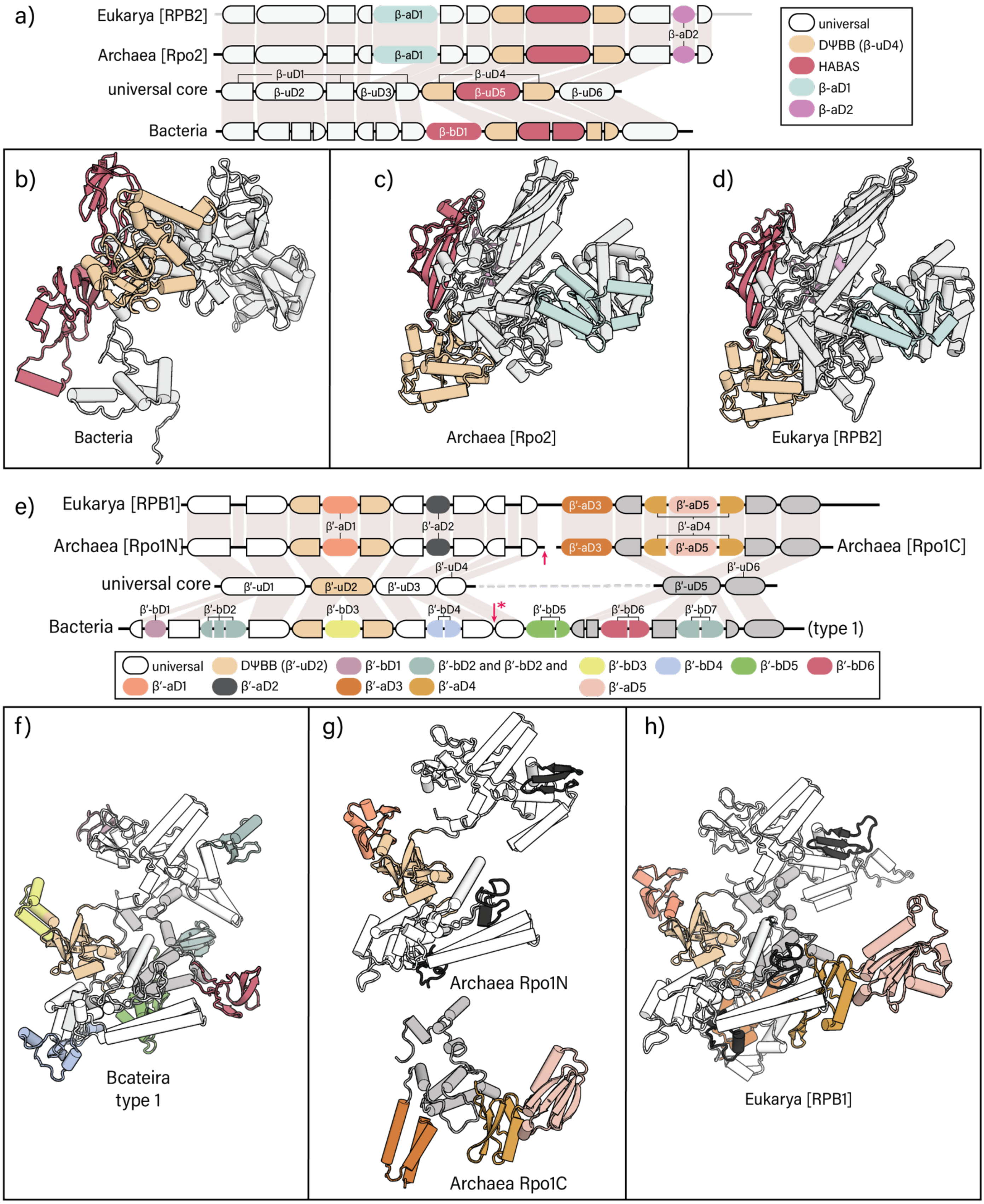

Figure S3. Multi-domain organizations of RNAP-β in bacteria, archaea and eukarya. (a) Domain organization of RNAP-β. First row: domains of eukaryotic RNAP-β. Second row: domains of archaeal RNAP-β. Third row: universally shared domains between archaeal and bacterial orthologs. Fourth row: domains of bacterial type 1 RNAP-β. (b-d) Three-dimensional structure of the RNAP-β orthologs. Domains are coloured as in (a). (e) Domain organization of RNAP-β’. First row: domains of eukaryotic RNAP-β’. Second row: domains of archaeal RNAP-β’. Third row: universally shared domains between archaeal and bacterial orthologs. Fourth row: domains of bacterial type 1 RNAP-β’. (b-d) Three-dimensional structure of the RNAP-β’ orthologs. Domains are coloured as in (e).

Figure S4

Pairwise sequence and structure comparisons of BEAN domains

Query: RNAP-B’-uD5 Target: RNAP-B type 4 (1)

Probab=97.60
E-value=3.6e-12
Score=54.78
Aligned_cols=43
Identities=19%
Similarity=0.288
Sum_probs=40.3
Template_Neff=5.900

```
Q RNAP-B’-uD5        SKTITGRVARNNIVDLVKDEVIIRENDLITEEKAKKIEALGYEKI
Q Consensus          ~eRi~GR~~aedv~~p~tg~viv~~g~~I~~~~a~~Ie~agi~~V
                     .+++.|+.+..||.  +||+|++++|..||...+++|.++|++.|
T Consensus          pe~l~G~~~~~Di~--~tGeviv~~G~kiT~r~ikkL~~~gi~~i
T RNAP-B-type4 (1) PERLRGETASFDIE--ANGKVYVEKGRRITARHIRQLEKDDVKLI
Confidence           48999999999999  7999999999999999999999999865
```

SSAP score: 88.27
Aligned residues: 43
Percentage overlap: 86
Percentage identity: 16
RMSD: 2.04

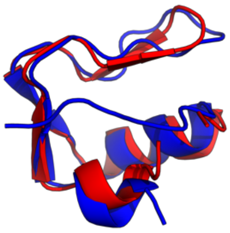

Query: RNAP-B’-uD5 Target: RNAP-B type 4 (2)

Probab=98.71
E-value=2.3e-16
Score=71.60
Aligned_cols=46
Identities=22%
Similarity=0.320
Sum_probs=43.9
Template_Neff=6.800

```
Q RNAP-B’-uD5        KTITGRVARNNIVDLVKDEVIIRENDLITEEKAKKIEALGYEKIKVR
Q Consensus          eRi~GR~~aedv~~p~tg~viv~~g~~I~~~~a~~Ie~agi~~V~VR
                     +.++||++++||.||. |++++.+|+.||++.+++|.++||++|.|-
T Consensus          e~l~G~~la~Divd~~-GEvl~eag~~it~~~l~~l~~~gi~~v~v~
T RNAP-B-type4 (2) EYIAGKVVAKDYIDES-GELICAANMELSLDLLAKLSQSGHKRIETL
Confidence           678999999999998 99999999999999999999999999875
```

SSAP score: 92.47
Aligned residues: 50
Percentage overlap: 96
Percentage identity: 20
RMSD: 2.13

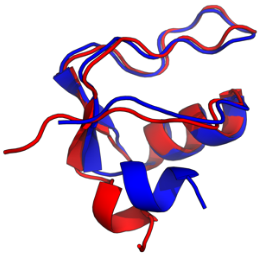

Query: RNAP-B’-uD5 Target: RNAP-B type 3

Probab=98.55
E-value=1.5e-15
Score=69.57
Aligned_cols=45
Identities=20%
Similarity=0.321
Sum_probs=42.3
Template_Neff=4.600

```
Q RNAP-B’-uD5        TGRVARNNIVDLVKDEVIIRENDLITEEKAKKIEALGYEKIKVRSP
Q Consensus          ~GR~~aedv~~p~tg~viv~~g~~I~~~~a~~Ie~agi~~V~VRS~
                     +|+.+|+|+.||. |++++++|+.||++.+.+|+.+||.+|+|.--
T Consensus          ~g~~lAe~ivDpe-GEilvekGt~i~r~~~~~le~~~iq~v~V~~~
T RNAP-B-type3       FNQILAEPIVDPE-GEVIAEKGEKLERKLLDKLEEITIQSVKIINE
Confidence           5899999999998 99999999999999999999999999998754
```

SSAP score: 89.84
Aligned residues: 47
Percentage overlap: 94
Percentage identity: 14
RMSD: 3.66

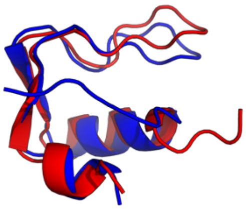

Query: RNAP-B’-uD5 Target: RNAP-B type 2 (2)

<table>
<tr><td>Probab=93.03
E-value=3.4e-07
Score=32.52
Aligned_cols=33
Identities=30%
Similarity=0.421
Sum_probs=28.6
Template_Neff=1.400</td><td>Q RNAP-B’-uD5 ARNNIVDLVKDEVIIRENDLITEEKAKKIEALGY
Q Consensus ~aedv~~p~tg~viv~~g~~I~~~~a~~Ie~agi
+..||.|-.||+++..+|..++.++++.| .-|+
T Consensus ~~rdvr~l~tg~lia~~g~rv~~e~a~~i-~ig~
T RNAP-B-type2 (2) TTRDVRHLSTGELIARAGARVDAELAKII-AIGV
Confidence 5678999999999999999999999988 4543</td></tr>
<tr><td>SSAP score: 91.09
Aligned residues: 41
Percentage overlap: 82
Percentage identity: 16
RMSD: 0.91</td><td>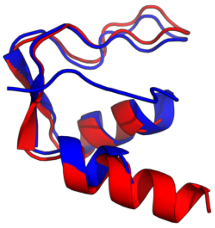</td></tr>
<tr><td colspan="2">Query: RNAP-B’-uD5 Target: RNAP-B type 2 (1)</td></tr>
<tr><td>Probab=98.40
E-value=6.3e-15
Score=66.97
Aligned_cols=45
Identities=27%
Similarity=0.392
Sum_probs=42.9
Template_Neff=4.300</td><td>Q RNAP-B’-uD5 KTITGRVARNNIVDLVKDEVIIRENDLITEEKAKKIEALGYEKIKVR
Q Consensus eRi~GR~~aedv~~p~tg~viv~~g~~I~~~~a~~Ie~agi~~V~VR
.+++||++.+||+|| |++|+++|+.||.+++++|.+.+++.|.||
T Consensus ~~lvg~ilr~dI~dp--gelI~~aG~~It~~~ak~ia~~~~~~I~V~
T RNAP-B-type2 (1) LWLNGQLLREDIVNP--GDLIAEAGSEIDRALATKIADLPLRAIKIH
Confidence 579999999999988 8999999999999999999999999999986</td></tr>
<tr><td>SSAP score: 88.13
Aligned residues: 45
Percentage overlap: 90
Percentage identity: 16
RMSD: 2.82</td><td>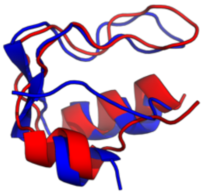</td></tr>
<tr><td colspan="2">Query: RNAP-B’-uD5 Target: uL10a</td></tr>
<tr><td>Probab=96.23
E-value=2.2e-09
Score=43.27
Aligned_cols=41
Identities=24%
Similarity=0.413
Sum_probs=37.9
Template_Neff=4.200</td><td>Q RNAP-B’-uD5 GRVARNNIVDLVKDEVIIRENDLITEEKAKKIEALGYEKIK
Q Consensus GR~~aedv~~p~tg~viv~~g~~I~~~~a~~Ie~agi~~V~
|..+.+||.-|....+++++|+.|+.+++..|...||.=+.
T Consensus G~iAp~DIvIpa~dt~v~k~Ge~Is~~lA~~L~kL~I~P~e
T uL10a GDVASMDVVVTAKDTTPVKKGEVINEKLASMLSKLDIKPVE
Confidence 67899999999999999999999999999999999997554</td></tr>
<tr><td>SSAP score: 87.77
Aligned residues: 41
Percentage overlap: 82
Percentage identity: 14
RMSD: 1.42</td><td>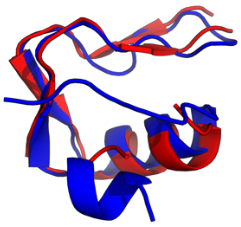</td></tr>
<tr><td colspan="2">Query: RNAP-B’-uD5 Target: PUF</td></tr>
<tr><td>Probab=96.07
E-value=3.5e-09
Score=42.87
Aligned_cols=46
Identities=17%
Similarity=0.166
Sum_probs=40.7
Template_Neff=4.100</td><td>Q RNAP-B’-uD5 KTITGRVARNNIVDLVKDEVIIRENDLITEEKAKKIEALGYEKIKVR
Q Consensus eRi~GR~~aedv~~p~tg~viv~~g~~I~~~~a~~Ie~agi~~V~VR
+---|-+++.+|.+|. |+++..+|..+|+..+.+|.+.||..|.|.
T Consensus ~L~~g~~Lak~iy~~~-G~iLL~~Gv~Lt~~~I~~Lk~~Gi~~vYIe
T PUF ILTPGMVLAKPIYDAQ-GKLLIDRNIKLKKLYIDKLIQYKIEAVYIS
Confidence 3345777899999987 9999999999999999999999999999873</td></tr>
</table>

<table>
<tr><td>SSAP score: 90.06
Aligned residues: 48
Percentage overlap: 87
Percentage identity: 18
RMSD: 1.92</td><td>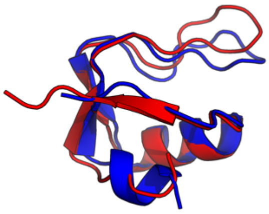</td></tr>
<tr><td colspan="2">Query: RNAP-B’-uD5 Target: MoeA</td></tr>
<tr><td>Probab=97.82
E-value=7.7e-13
Score=57.74
Aligned_cols=47
Identities=23%
Similarity=0.423
Sum_probs=43.1
Template_Neff=6.300</td><td>Q RNAP-B’-uD5 KTITGRVARNNIVDLVKDEVIIRENDLITEEKAKKIEALGYEKIKVRS
Q Consensus eRi~GR~~aedv~~p~tg~viv~~g~~I~~~~a~~Ie~agi~~V~VRS
+.-+||++++||..|. |++++++|+.|++..+..|...|+++|.|+.
T Consensus ~~a~Grvlaedv~a~~-G~~il~~G~~i~p~~i~~Las~Gi~~V~V~~
T MoeA IDAYSRILYEDIISNK-DEIILKAGKILEASDIGVIAALGFTSVKVFK
Confidence 3458999999999987 9999999999999999999999999999863</td></tr>
<tr><td>SSAP score: 90.59
Aligned residues: 49
Percentage overlap: 90
Percentage identity: 18
RMSD: 1.99</td><td>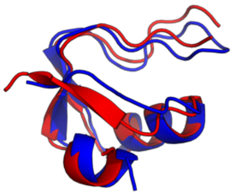</td></tr>
<tr><td colspan="2">Query: RNAP-B’-uD5 Target: Ferredoxin</td></tr>
<tr><td>Probab=97.82
E-value=8e-13
Score=58.42
Aligned_cols=42
Identities=19%
Similarity=0.395
Sum_probs=38.4
Template_Neff=2.100</td><td>Q RNAP-B’-uD5 KTITGRVARNNIVDLVKDEVIIRENDLITEEKAKKIEALGYE
Q Consensus eRi~GR~~aedv~~p~tg~viv~~g~~I~~~~a~~Ie~agi~
+-++||-+..|+.||.||++++.+|.+||+..+.++++..++
T Consensus e~LiGrRaeqnilHPi~G~iLVe~gklITRs~i~qL~dt~~~
T Ferredoxin ETLIGRRAEQDILHPLEGKILVEAGKLITRSMISQLKDTRTS
Confidence 568999999999999999999999999999999999876553</td></tr>
<tr><td>SSAP score: 86.81
Aligned residues: 50
Percentage overlap: 89
Percentage identity: 22
RMSD: 7.72</td><td>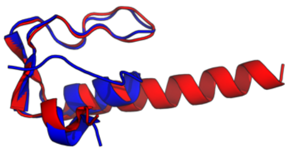</td></tr>
<tr><td colspan="2">Query: RNAP-B’-uD5 Target: OKR decarboxylase</td></tr>
<tr><td>Probab=82.24
E-value=1e-05
Score=27.63
Aligned_cols=38
Identities=26%
Similarity=0.240
Sum_probs=30.6
Template_Neff=5.100</td><td>Q RNAP-B’-uD5 27 KTITGRVARNNIVDLVKDEVIIRENDLITEEKAKKIEA 64 (81)
Q Consensus 27 eRi~GR~~aedv~~p~tg~viv~~g~~I~~~~a~~Ie~ 64 (81)
+...||++++-|.-===|=-++-.|+.||.+.++.|..
T Consensus 8 ~ea~GrI~ae~i~pYPPGIP~l~PGE~it~e~i~yl~~ 45 (52)
T OKR decarboxylase 8 GEAVGRVAAEAVMIYPPGIPICIPGELITAETIEEILY 45 (52)
Confidence 45689999998643347788899999999999988854</td></tr>
<tr><td>SSAP score: 84.33
Aligned residues: 48
Percentage overlap: 87
Percentage identity: 14
RMSD: 5.27</td><td>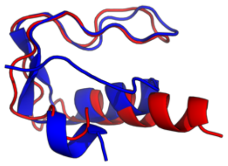</td></tr>
<tr><td colspan="2">Pairwise sequence and structure comparisons of HABAS domains</td></tr>
</table>

Query: RNAP-B-bD1 Target: RNAP-B’ type 4

Probab=92.99
E-value=3.4e-07
Score=40.79
Aligned_cols=57
Identities=18%
Similarity=0.105
Sum_probs=33.0
Template_Neff=5.200

```
Q RNAP-B-bD1      PITKVNGTVSYVDANEIV--------VKDDHGNEHFH-----YLQKYQRSNQDTCLNQRPIVKIGDKVISGQVLAD
Q Consensus       v~A~~~G~V~~Vda~~I~--------V~~~~g~~~~Y-----~L~kF~RSNq~TcinQrPiV~~G~~VkkG~vLAD
                  +.|+++|+|.+.+-+.|.        +... |+....     ++.+|.-     -+--+-.|+-||.|+|||+||.
T Consensus       i~a~~~G~v~~~nl~~V~~~~G~~iv~~Rn-gei~I~D~~gre~ery~v-----pyGa~l~v~dG~~V~kg~~lae
T RNAP-B’-type4   VESNFEGIVRIRNRNVARNSEGDLVVMARN-LAVVIVDVDGTERAVNRV-----QYGARLKVDEGDTIKRGQRIAE
Confidence        568899998887654332        2211 222111     2222222     2233446899999999999985
```

SSAP score: 83.88
Aligned residues: 59
Percentage overlap: 70
Percentage identity: 20
RMSD: 3.08

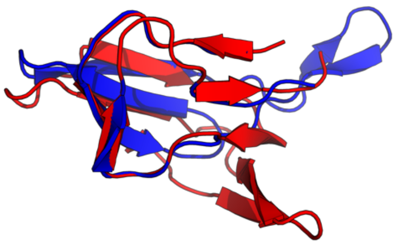

Query: RNAP-B’ type 4 Target: RNAP-B’-bD6

Probab=97.07
E-value=7e-11
Score=58.28
Aligned_cols=57
Identities=32%
Similarity=0.557
Sum_probs=42.1
Template_Neff=5.800

```
Q RNAP-B’-type4   YVESNFEGIVRIRNRNVARNSEGDLVVMARNLAVVIVDVDGTERAVNRVQYGARLKVDEGDTIKRGQRIAE
Q Consensus       ~i~a~~~G~v~~~nl~~V~~~~G~~iv~~Rngei~I~D~~gre~ery~vpyGa~l~v~dG~~V~kg~~lae
                  .+-|..+|+|+|...      +|..       .|.|.+++| +...|.||+|++|.|++||.|++||.|.+
T Consensus       Aiiaei~G~V~~~~~~------~g~r-------~I~I~~~~~-e~~eY~ip~~~~l~V~~Gd~V~~G~~Lte
T RNAP-B’-bD6     AVVVEVNGKVRFGEN------KGRR-------EVEVLG-NG-EVGTYPVHYGSRLKFEEGTEVEIGDALTE
Confidence        456788888877543      2221       234444444 57799999999999999999999999975
```

SSAP score: 87.14
Aligned residues: 63
Percentage overlap: 75
Percentage identity: 25
RMSD: 2.82

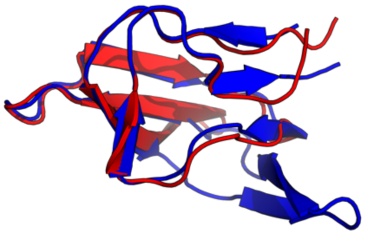

Query: psd (2) Target: RNAP-B’-bD6

Probab=77.71
E-value=1.9e-05
Score=27.74
Aligned_cols=37
Identities=16%
Similarity=0.216
Sum_probs=24.1
Template_Neff=5.800

```
Q psd (2)           NVLLMESPAGKMAFQQVAGLVARRIVCRINEGTVLQRGERF
Q Consensus         ~~i~i~t~~g~~~v~QIAG~iARRIv~~v~~Gd~v~~Ger~
                    ..|.|..+.|+..--.|    -.+-.-.|+.||.|++||++
T Consensus         r~I~I~~~~~e~~eY~i----p~~~~l~V~~Gd~V~~G~~L
T RNAP-B’-bD6       REVEVLG-NGEVGTYPV----HYGSRLKFEEGTEVEIGDAL
Confidence          44555555554433332    34456689999999999986
```

SSAP score: 79.82
Aligned residues: 62
Percentage overlap: 72
Percentage identity: 12
RMSD: 3.09

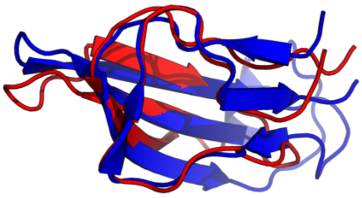

Query: peptidase M23 Target: RNAP-B’-bD6

Probab=79.91
E-value=1.4e-05
Score=27.94
Aligned_cols=35
Identities=17%
Similarity=0.259
Sum_probs=25.0
Template_Neff=5.800

```
Q peptidase M23     YYVIIEQSSGLFCKYGE--LADFTVRKGDKIEAGNLI
Q Consensus         ~~iiv~~~sG~~cKYgE--la~~~v~kG~~I~~~~lI
                    +.|+|.++.|-.-.|-=  =+...|+.||.|++||++
T Consensus         r~I~I~~~~~e~~eY~ip~~~~l~V~~Gd~V~~G~~L
T RNAP-B’-bD6       REVEVLG-NGEVGTYPVHYGSRLKFEEGTEVEIGDAL
Confidence          45677777776655532  245679999999999986
```

SSAP score: 83.09
Aligned residues: 60
Percentage overlap: 81
Percentage identity: 17
RMSD: 3.45

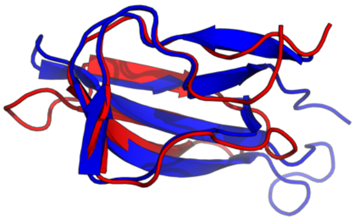

Query: RND Target: BCC

Probab=99.64
E-value=5.7e-25
Score=105.73
Aligned_cols=63
Identities=38%
Similarity=0.586
Sum_probs=59.9
Template_Neff=2.800

```
Q RND             TLAFKIGGIVNRVLVKEGDKVKKGQLLATLNLS----EIHAPESGYVLRKFVNSGQVVSIGDPILLT
Q Consensus       ~i~~~v~G~I~~i~v~~Gd~V~~Gq~La~ld~~----~I~AP~~GvV~~~~v~~G~~v~~g~~l~~i
                  .+.||.+|+|.+++|++||+||+||+|+.|++.    .|.||+||+|.++.|++||.|+.|++|..|
T Consensus       ~V~APmpG~ilki~Vk~Gd~Vk~G~~lliLEAMKMENeI~aP~~G~Vk~I~VkeG~~V~tGd~Lvvi
T BCC             VVSAPMPGKVLRVLVRVGDRVRVGQGLLVLEAMKMENEIPSPRDGVVKRILVKEGEAVDTGQPLIEL
Confidence        5789999999999999999999999999975    699999999999999999999999875
```

SSAP score: 93.23
Aligned residues: 66
Percentage overlap: 94
Percentage identity: 36
RMSD: 1.24

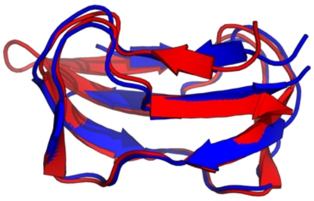

Query: RND Target: OGDC-E2

Probab=98.96
E-value=7.8e-18
Score=78.73
Aligned_cols=59
Identities=19%
Similarity=0.210
Sum_probs=55.0
Template_Neff=5.000

```
Q RND             IGGIVNRVLVKEGDKVKKGQLLATLNL----SEIHAPESGYVLRKFVNSGQVVSIGDPILLTN
Q Consensus       v~G~I~~i~v~~Gd~V~~Gq~La~ld~----~~I~AP~~GvV~~~~v~~G~~v~~g~~l~~i~
                  ..|.|.++++++||+|++||+|+++.+    ..|-||.+|++.++.+++|+.|..|++|+.|.
T Consensus       ~eg~i~~Wl~~~GD~V~~~e~l~eveTDKa~~Ev~ap~~G~l~~i~~~eG~~V~vG~~i~~i~
T OGDC-E2         ADATVATWHKKPGDAVVRDEVLVEIETDKVVLEVPASADGILDAVLEDEGTTVTSRQILGRLR
Confidence        578999999999999999999975    5688999999999999999999999874
```

SSAP score: 90.19
Aligned residues: 68
Percentage overlap: 83
Percentage identity: 16
RMSD: 1.23

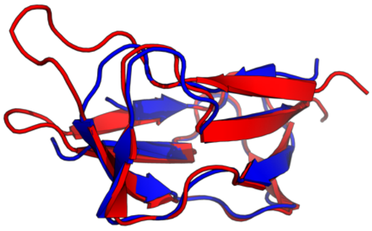

Query: RNAP-B’-bD6 Target: RNAP-B’ type 2

Probab=96.60
E-value=5.9e-10
Score=43.73
Aligned_cols=30
Identities=20%
Similarity=0.245
Sum_probs=27.3
Template_Neff=5.000

```
Q RNAP-B’-bD6     GEVGTYPVHYGSRLKFEEGTEVEIGDALTE
Q Consensus       ~e~~eY~ip~~~~l~V~~Gd~V~~G~~Lte
                  ....+|.+|.+..+.|++||.|+.||.|+.
T Consensus       ~~~dey~lp~g~~i~Vk~G~~V~~g~vLa~
T RNAP-B’-type2   VYQDEYELPSGWKTQVQSGQWVDSGMVLAS
Confidence        345699999999999999999999999985
```

SSAP score: 74.80
Aligned residues: 34
Percentage overlap: 47
Percentage identity: 9
RMSD: 2.98

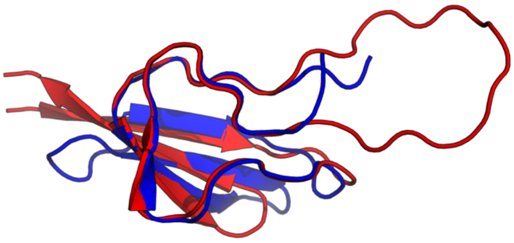

Query: RNAP-B’-bD6 Target: RNAP-B’ type 3

Probab=94.76
E-value=5.4e-08
Score=48.71
Aligned_cols=55
Identities=29%
Similarity=0.412
Sum_probs=40.7
Template_Neff=2.400

```
Q RNAP-B’-bD6   VVVEVNGKVRFGENKGRR-----------------EVEVLG-NGEVGTYPVHYGSRLKFEEGTEVEIGDALTE
Q Consensus     iiaei~G~V~~~~~~g~r-----------------~I~I~~~~~e~~eY~ip~~~~l~V~~Gd~V~~G~~Lte
                +=||++|+|++...-..|                 .|.+.+ .++..+|.+++|..|.|.+|+.|++|+.|.+
T Consensus     vra~~~g~v~~~~~l~tR~~RTrhGeea~q~e~~g~l~l~~-~~e~~~i~vt~GS~l~v~~G~~Ve~g~~laE
T RNAP-B’-type3 VRAASAGTVRYSKKLRLRSYRTRHGEDAQYVEMNGNISLDV-GNESGDIAVTQGSTLYVVDGQKVKKGQLLAE
Confidence      447888988885421111                 233333 4567799999999999999999999999875
```

SSAP score: 83.71
Aligned residues: 63
Percentage overlap: 76
Percentage identity: 17
RMSD: 3.53

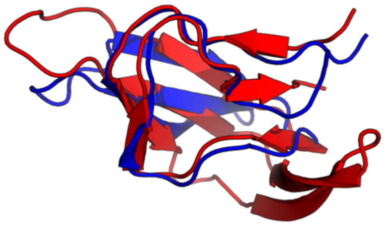

Query: RNAP-B’-bD6 Target: psd (1)

Probab=97.84
E-value=7.2e-13
Score=56.03
Aligned_cols=56
Identities=18%
Similarity=0.202
Sum_probs=46.0
Template_Neff=4.600

```
Q RNAP-B’-bD6      QAVVVEVNGKVRFGENKGR-REVEVLG-NGEVGTYPVHYGSRLKFEEGTEVEIGDALT
Q Consensus        ~Aiiaei~G~V~~~~~~g~-r~I~I~~~~~e~~eY~ip~~~~l~V~~Gd~V~~G~~Lt
                   ..++||.||+|...+.... .+|.|- -+|...+..+|.+..+.|+.||+|.+|+.+.
T Consensus        ~~vvsPADGkV~~i~~~~~~~~isIF-MsGSRvDv~lP~~~~~~V~~Gdkv~aGeTii
T psd (1)          DAVIAPADGRVVAIREMEPVRMVSIF-MNGSRVDLFFPMDAEISVKLGEMTHSGVTQM
Confidence         4689999999988887753 345554 4677889999999999999999999998653
```

SSAP score: 84.56
Aligned residues: 60
Percentage overlap: 82
Percentage identity: 12
RMSD: 2.75

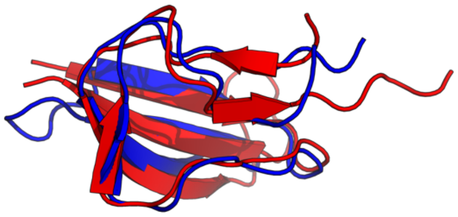

Query: RND Target: RsxC

Probab=97.62
E-value=3.1e-12
Score=54.42
Aligned_cols=43
Identities=37%
Similarity=0.389
Sum_probs=36.8
Template_Neff=4.700

```
Q RND              GIVNRVLVKEGDKVKKGQLLATLNL---SEIHAPESGYVLRKFVNS
Q Consensus        G~I~~i~v~~Gd~V~~Gq~La~ld~---~~I~AP~~GvV~~~~v~~
                   |.--+-.|++||+|++||.|++=+.   ..|.||++|+|.++.-.+
T Consensus        Gap~~p~Vk~GD~V~~Gq~Ia~~~g~vsapvHssvSG~V~~I~~~~
T RsxC             GAEGELCVSVGDKVLRGQPLTRGRGKM-LPVHAPTSGTVTAIAPHS
Confidence         4445678999999999999999887   789999999876554
```

SSAP score: 86.50
Aligned residues: 68
Percentage overlap: 90
Percentage identity: 25
RMSD: 5.23

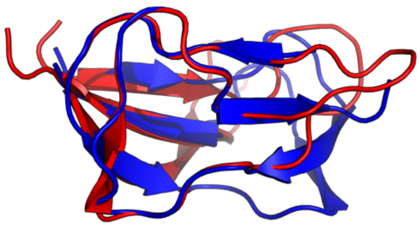

Query: RNAP-B-uD5 Target: RNAP-B-bD1

Probab=79.39
E-value=1.5e-05
Score=31.62
Aligned_cols=21
Identities=38%
Similarity=0.591
Sum_probs=17.9
Template_Neff=5.355

```
Q RNAP-B-uD5       NLDEMGIIRIGAFVESGDILV
Q Consensus        ~LDe~GIvriGaeV~~GDILV
                   -+...=+|.+|..|++||+|.
T Consensus        cinQrPiV~~Gd~VkkG~vLA
T RNAP-B-bD1       CLNQRPIVKIGDKVISGQVLA
Confidence         355667999999999999996
```

<table>
<tr><td>SSAP score: 64.80
Aligned residues: 46
Percentage overlap: 35
Percentage identity: 10
RMSD: 3.58</td><td>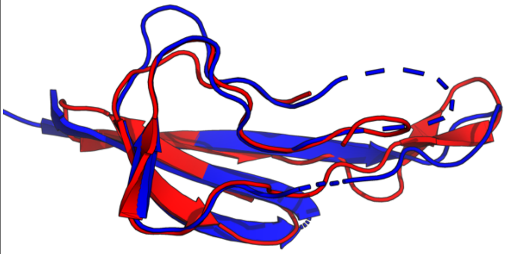</td></tr>
<tr><td colspan="2">Query: NusG Target: RNAP-B’-bD6</td></tr>
<tr><td>Probab=95.40
E-value=1.8e-08
Score=39.70
Aligned_cols=40
Identities=15%
Similarity=0.256
Sum_probs=30.7
Template_Neff=5.800</td><td>Q NusG   RVIFIETIDRRYTKTYYIPESAKVETGIKAGARIRQGMPLAKQ
Q Consensus   r~I~iET~~kkyskty~IPesa~~~~gikvG~rvr~g~plt~~
                |+|+|... ....+.|.||..+.|. ++.|++|+.|++|+..
T Consensus   r~I~I~~~-~~e~~eY~ip~~~~l~--V~~Gd~V~~G~~LteG
T Bp-bD6_ABI6963 REVEVLG--NGEVGTYPVHYGSRLK--FEEGTEVEIGDALTEG
Confidence   55666544 4455569999998886 8899999999999853</td></tr>
<tr><td>SSAP score: 81.20
Aligned residues: 44
Percentage overlap: 69
Percentage identity: 9
RMSD: 2.51</td><td>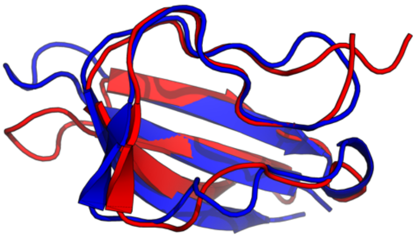</td></tr>
</table>

Figure S4. Pairwise comparisons of BEAN and HABAS domains. Scores derived from the pairwise comparison of BEAN and HABAS domains. Sequence comparisons were performed with HHalign (3). Structure comparisons were calculated with SSAP (4).

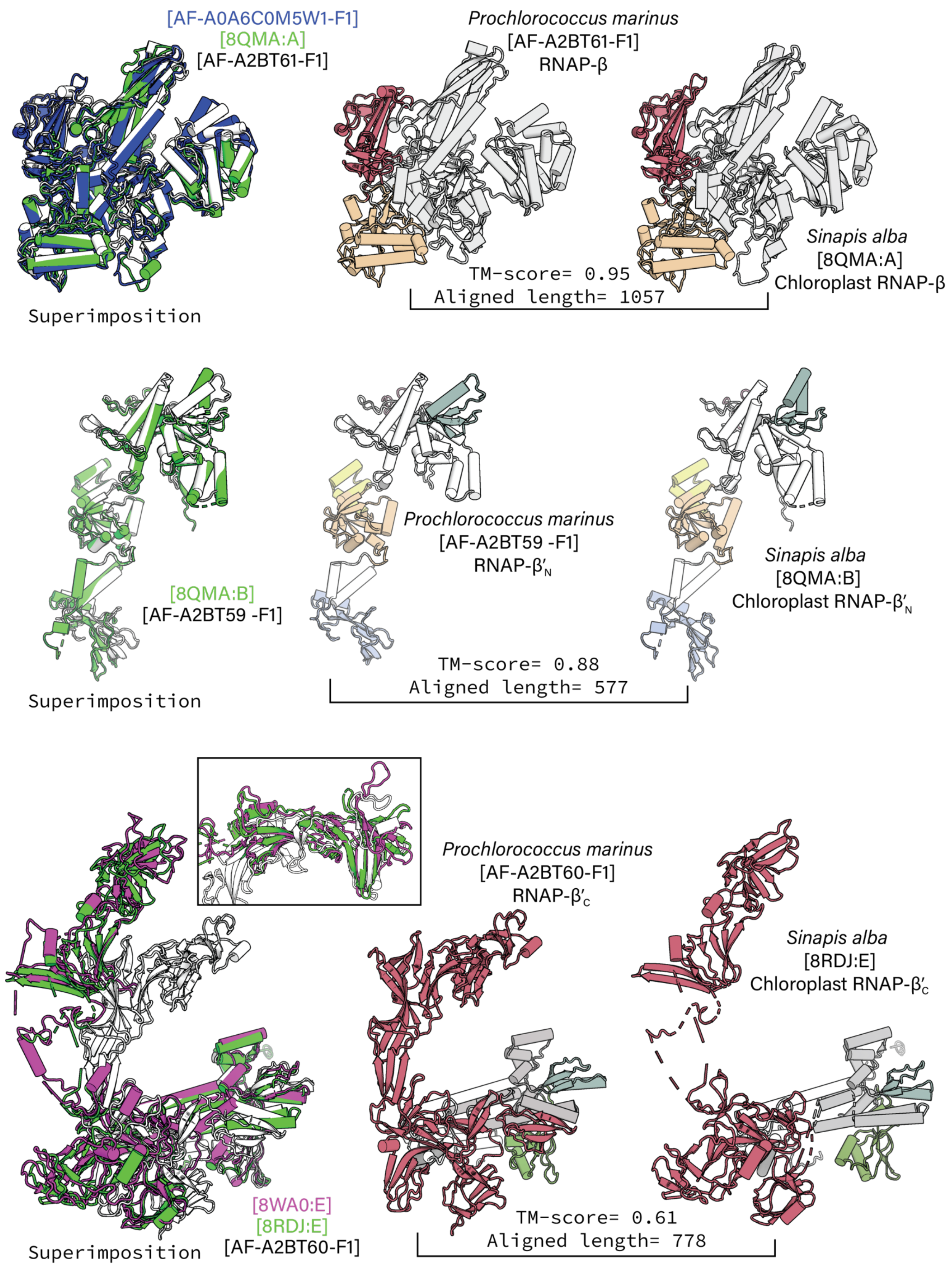

Figure S5. Structure comparisons of cyanobacteria and chloroplast RNAP subunits. Structure superimpositions were calculated with TM-align (8). Chloroplast RNAP-β and RNAP-β′$_N$ structures (1). Chloroplast RNAP-β′$_C$ structures (6; 7).

Figure S6

	Structure colored by pLDDT	Predicted Aligned Error
**Bacterial type 1 RNAP-β**<br>ID: AF-A2BT61-F1<br>Source: *Prochlorococcus marinus*	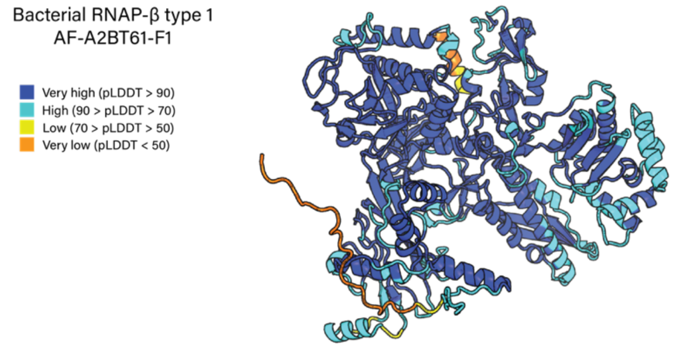	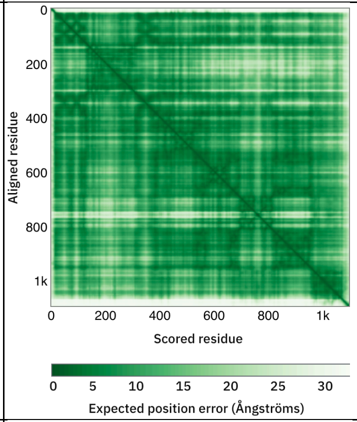
**Bacterial type 2 RNAP-β**<br>ID: AF-A9B6J3-F1<br>Source: *Herpetosiphon aurantiacus*	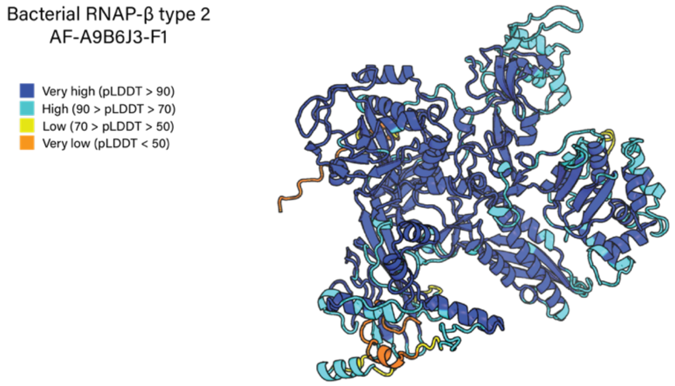	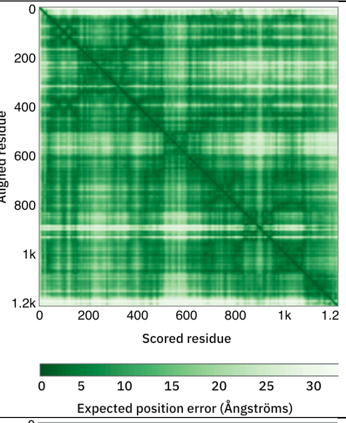
**Bacterial type 3 RNAP-β**<br>ID: AF-Q8ETY8-F1<br>Source: *Oceanobacillus iheyensis*	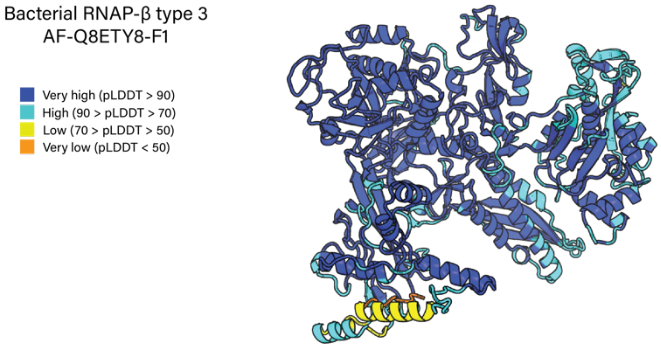	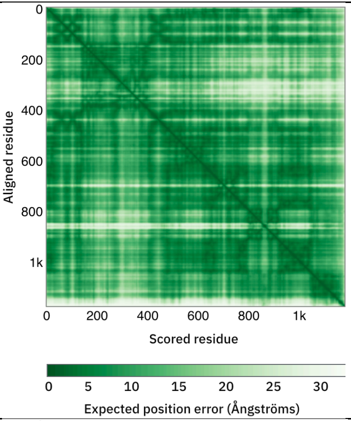
**Archaeal RNAP-β homolog**<br>ID: AF-P11513-F1<br>Source: *Sulfolobus acidocaldarius*	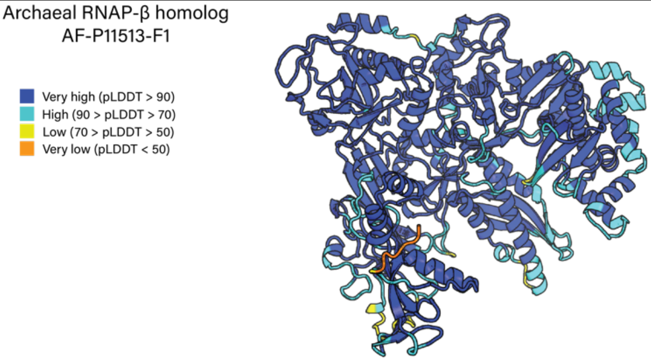	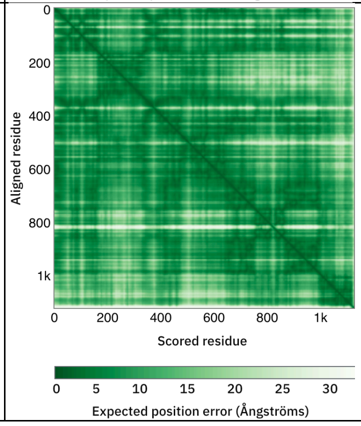

**Human RNAP-β homolog**<br>ID: AF-P30876-F1<br>Source: *Homo sapiens*	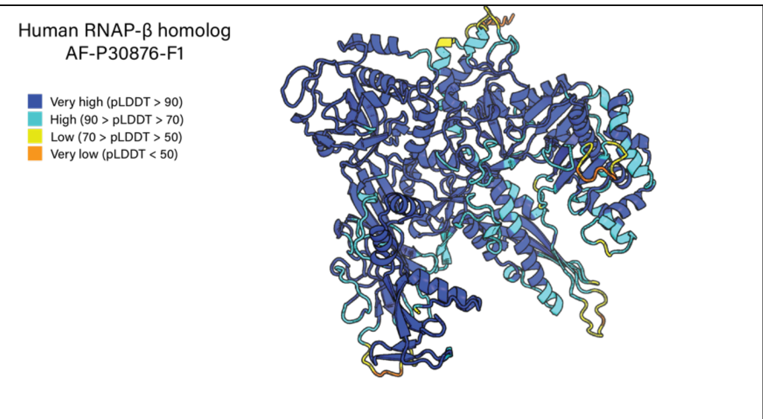	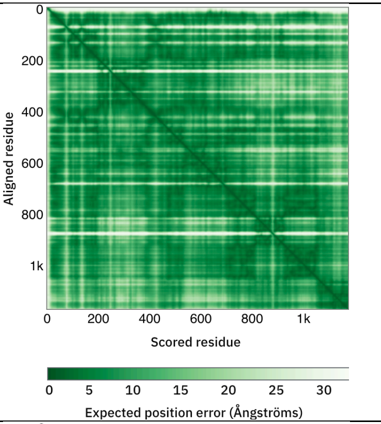
**Bacterial RNAP-β’ type 1**<br>ID: AF-Q0AUH3-F1<br>Source: *Syntrophomonas wolfei subsp. wolfei*	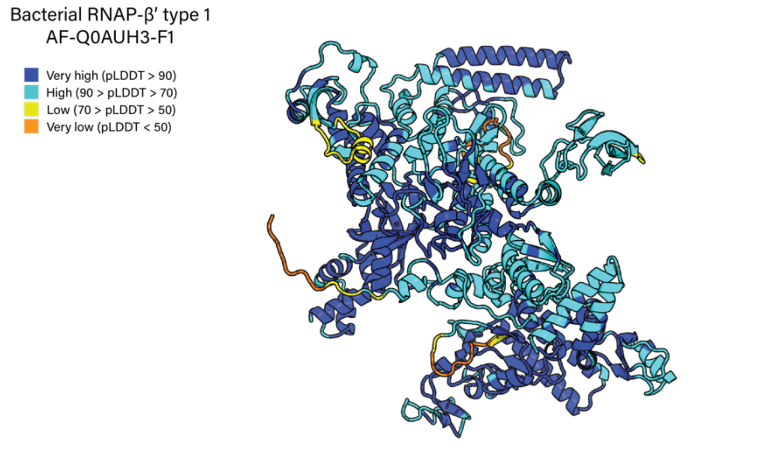	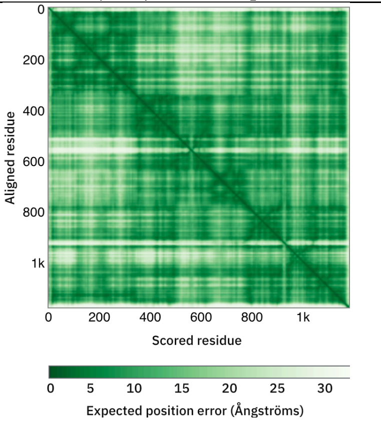
**Bacterial RNAP-β’ type 2**<br>ID: AF-Q3Z8V3-F1<br>Source: *Dehalococcoides mccartyi*	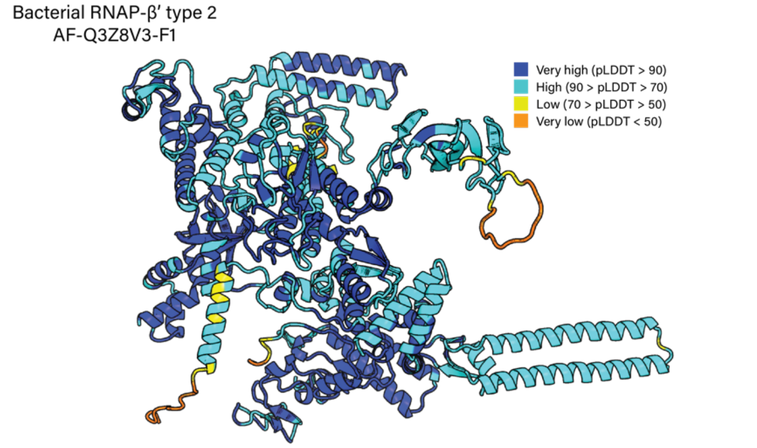	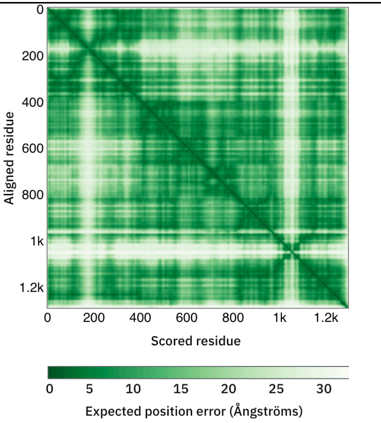
**Bacterial RNAP-β’$_N$ type 3**<br>ID: AF-A2BT59-F1<br>Source: *Prochlorococcus marinus*	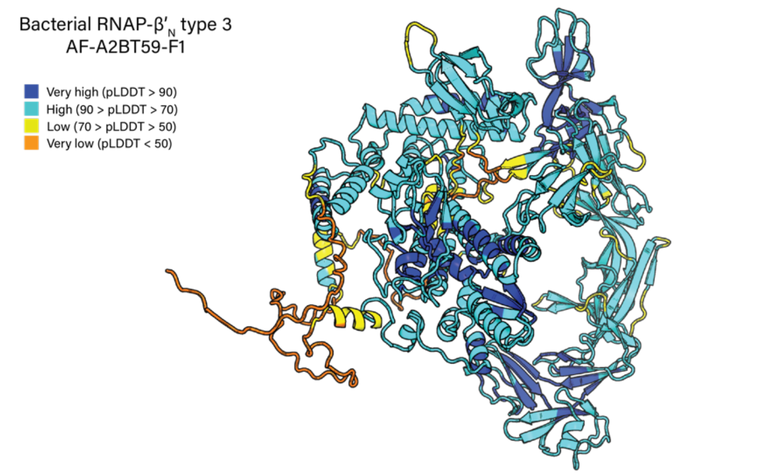	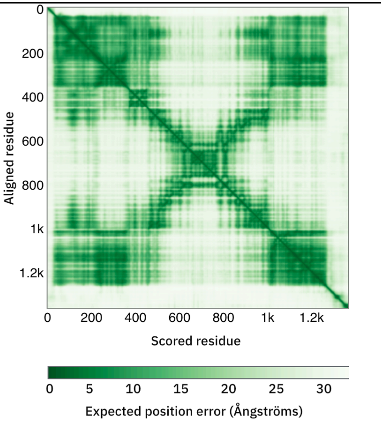

<table>
<tr>
<td>Bacterial RNAP-β'_C type 3
ID: AF-A2BT60-F1
Source: Prochlorococcus marinus</td>
<td>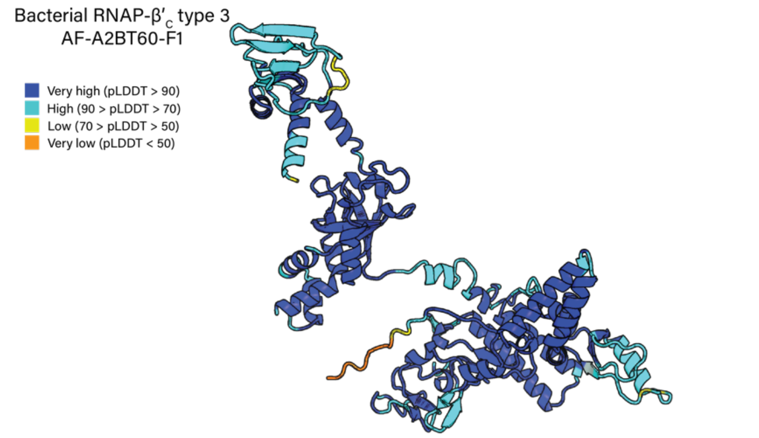
</td>
<td>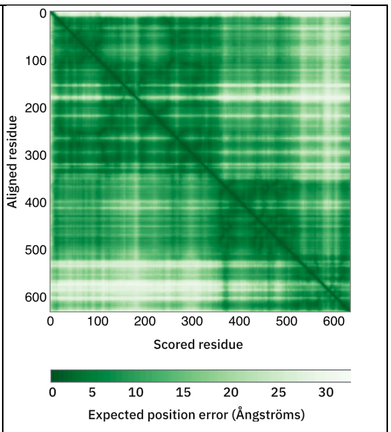
</td>
</tr>
<tr>
<td>Bacterial RNAP-β' type 4
ID: AF-A7IKQ1-F1
Source: Xanthobacter autotrophicus</td>
<td>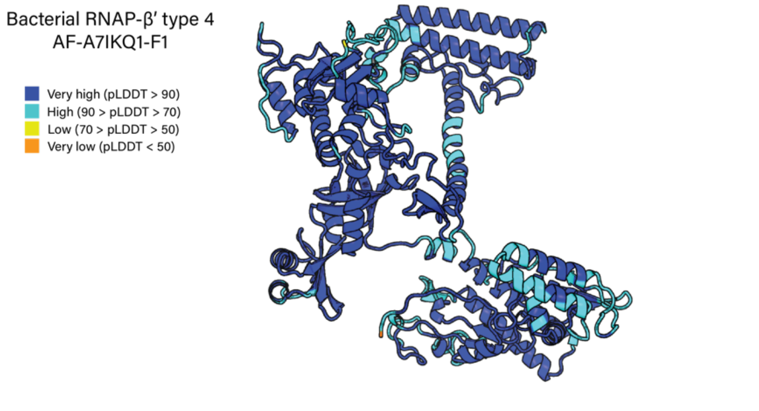
</td>
<td>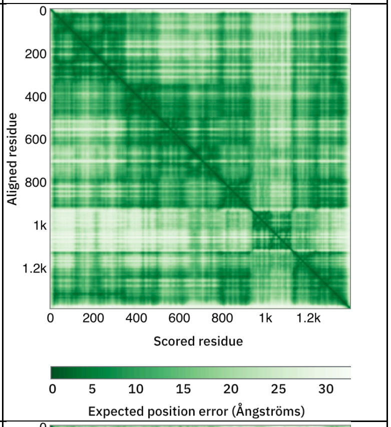
</td>
</tr>
<tr>
<td>Archaeal RNAP-β'_N homolog
ID: AF-P11512-F1
Source: Sulfolobus acidocaldarius</td>
<td>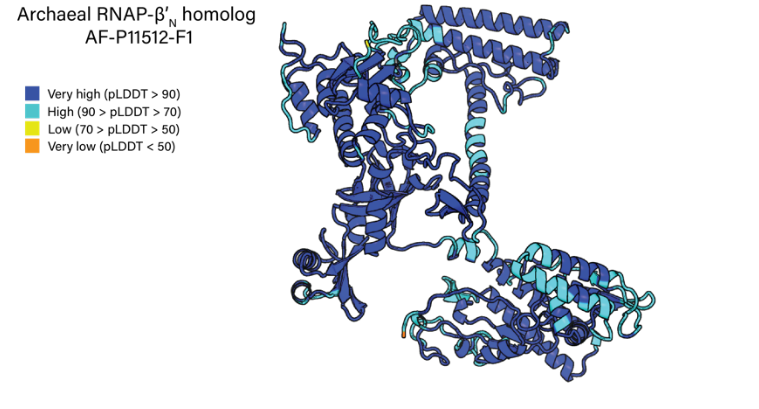
</td>
<td>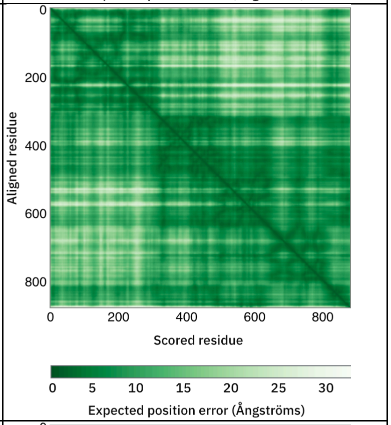
</td>
</tr>
<tr>
<td>Archaeal RNAP-β'C homolog
ID: AF-P11514-F1
Source: Sulfolobus acidocaldarius</td>
<td>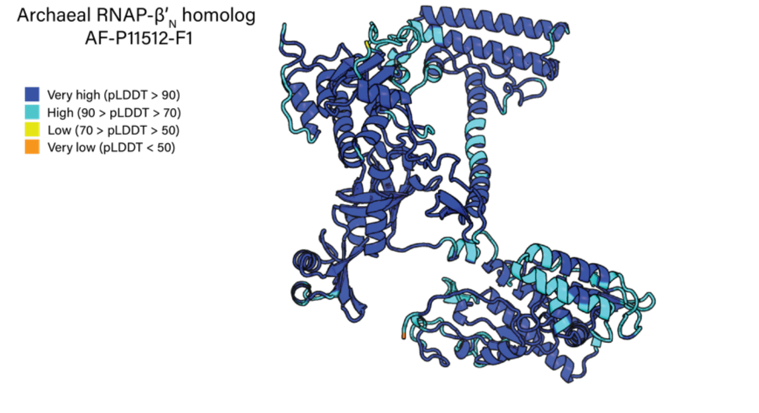
</td>
<td>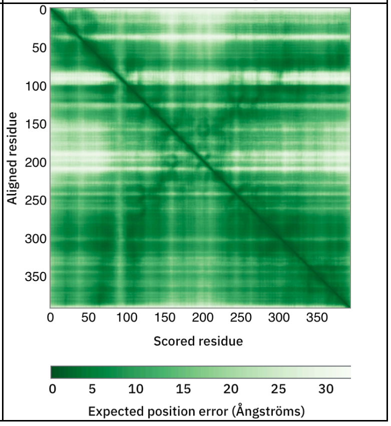
</td>
</tr>
</table>

Human RNAP-β’ homolog
ID: AF-P24928-F1
Source: *Homo sapiens*

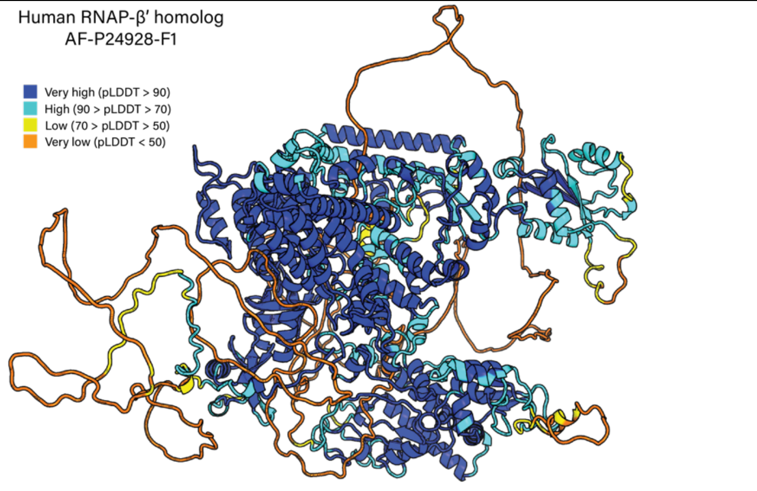

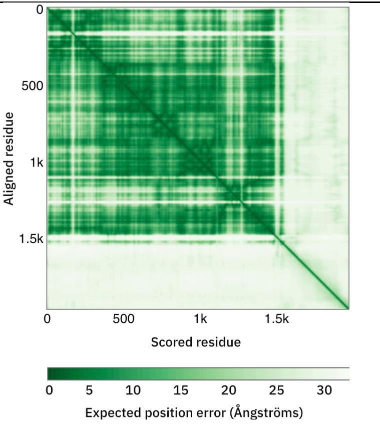

Figure S6. Structure predictions of RNAP subunits. Structure predictions were obtained from AlphaFold DB (5) and colored by predicted local distance difference test (pLDDT).

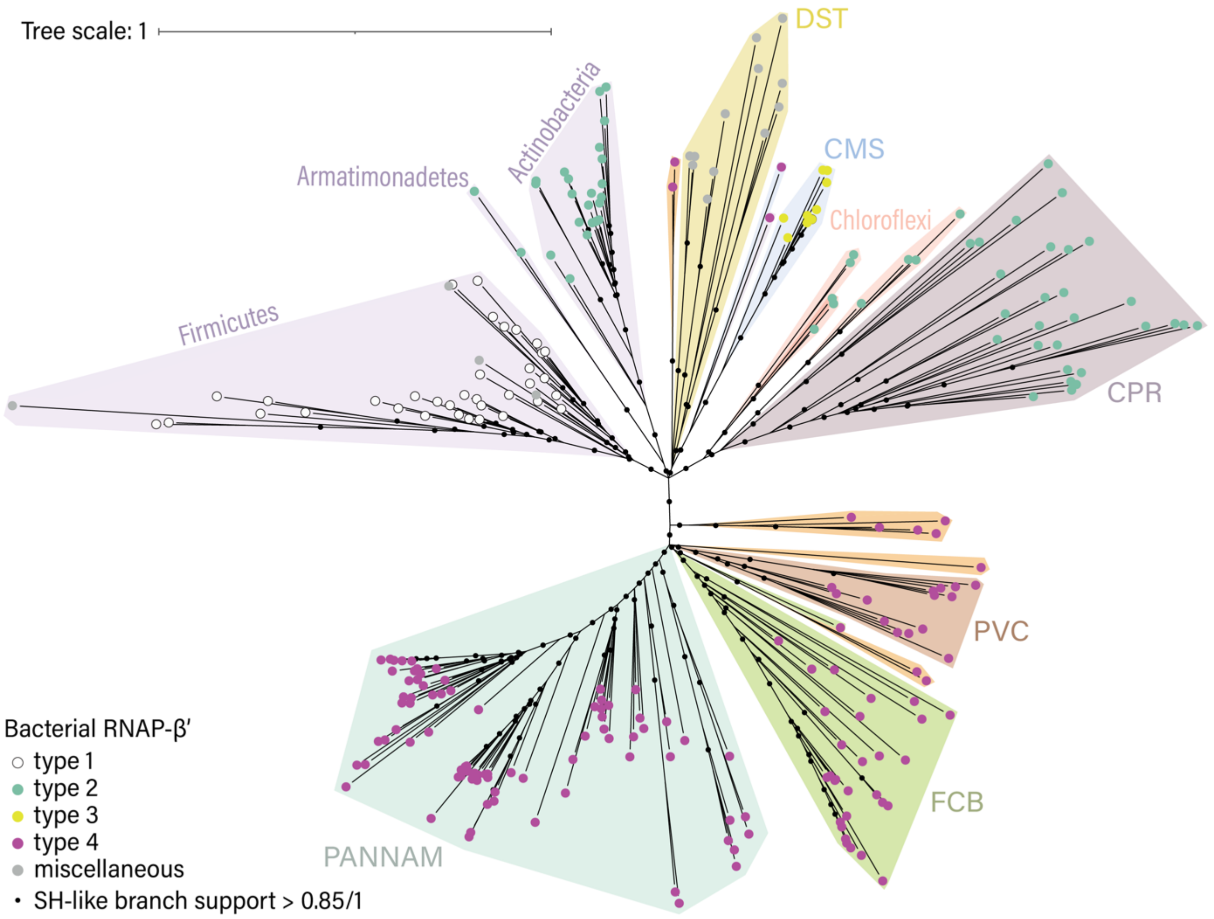

Figure S7. Maximum likelihood tree of RNAP-β'. The ML tree (inferred with model LG+G+I) of RNAP-β' was calculated with PhyML using positions conserved in at least 90% of sequences in the MSA. Type 3 RNAP-β'$_{BacN}$ and RNAP-β'$_{BacC}$ sequences are concatenated in the MSA.

References

1. do Prado PFV, Ahrens FM, Liebers M, Ditz N, Braun HP, Pfannschmidt T, Hillen HS (2024) Structure of the multi-subunit chloroplast RNA polymerase. Mol Cell 84:910-925.e915.
2. Frickey T, Lupas A (2004) CLANS: a Java application for visualizing protein families based on pairwise similarity. Bioinformatics 20:3702-3704.
3. Steinegger M, Meier M, Mirdita M, Vöhringer H, Haunsberger SJ, Söding J (2019) HH-suite3 for fast remote homology detection and deep protein annotation. BMC Bioinformatics 20:473.
4. Taylor WR, Orengo CA (1989) Protein structure alignment. J Mol Biol 208:1-22.
5. Varadi M, Anyango S, Deshpande M, Nair S, Natassia C, Yordanova G, Yuan D, Stroe O, Wood G, Laydon A, Žídek A, Green T, Tunyasuvunakool K, Petersen S, Jumper J, Clancy E, Green R, Vora A, Lutfi M, Figurnov M, Cowie A, Hobbs N, Kohli P, Kleywegt G, Birney E, Hassabis D, Velankar S (2022) AlphaFold Protein Structure Database: massively expanding the structural coverage of protein-sequence space with high-accuracy models. Nucleic Acids Res 50:D439-d444.
6. Vergara-Cruces Á, Pramanick I, Pearce D, Vogirala VK, Byrne MJ, Low JKK, Webster MW (2024) Structure of the plant plastid-encoded RNA polymerase. Cell 187:1145-1159.e1121.
7. Wu XX, Mu WH, Li F, Sun SY, Cui CJ, Kim C, Zhou F, Zhang Y (2024) Cryo-EM structures of the plant plastid-encoded RNA polymerase. Cell 187:1127-1144.e1121.
8. Zhang Y, Skolnick J (2005) TM-align: a protein structure alignment algorithm based on the TM-score. Nucleic Acids Res 33:2302-2309.
